# Overcoming uncollapsed haplotypes in long-read assemblies of non-model organisms

**DOI:** 10.1186/s12859-021-04118-3

**Published:** 2021-06-05

**Authors:** Nadège Guiglielmoni, Antoine Houtain, Alessandro Derzelle, Karine Van Doninck, Jean-François Flot

**Affiliations:** 1 Service Evolution Biologique et Ecologie, Université libre de Bruxelles (ULB), Avenue Franklin D. Roosevelt 50, 1050 Brussels, Belgium; 2 Laboratoire d’Ecologie et Génétique Evolutive, Université de Namur, Rue de Bruxelles 61, 5000 Namur, Belgium; 3 Département de Biologie des Organismes, Université libre de Bruxelles (ULB), Avenue Franklin D. Roosevelt 50, 1050 Brussels, Belgium; 4 Interuniversity Institute of Bioinformatics in Brussels - (IB)², Avenue Franklin D. Roosevelt 50, 1050 Brussels, Belgium

**Keywords:** Genome assembly, Long reads, Haplotype collapsing

## Abstract

**Background:**

Long-read sequencing is revolutionizing genome assembly: as PacBio and Nanopore technologies become more accessible in technicity and in cost, long-read assemblers flourish and are starting to deliver chromosome-level assemblies. However, these long reads are usually error-prone, making the generation of a haploid reference out of a diploid genome a difficult enterprise. Failure to properly collapse haplotypes results in fragmented and structurally incorrect assemblies and wreaks havoc on orthology inference pipelines, yet this serious issue is rarely acknowledged and dealt with in genomic projects, and an independent, comparative benchmark of the capacity of assemblers and post-processing tools to properly collapse or purge haplotypes is still lacking.

**Results:**

We tested different assembly strategies on the genome of the rotifer *Adineta vaga*, a non-model organism for which high coverages of both PacBio and Nanopore reads were available. The assemblers we tested (Canu, Flye, NextDenovo, Ra, Raven, Shasta and wtdbg2) exhibited strikingly different behaviors when dealing with highly heterozygous regions, resulting in variable amounts of uncollapsed haplotypes. Filtering reads generally improved haploid assemblies, and we also benchmarked three post-processing tools aimed at detecting and purging uncollapsed haplotypes in long-read assemblies: HaploMerger2, purge_haplotigs and purge_dups.

**Conclusions:**

We provide a thorough evaluation of popular assemblers on a non-model eukaryote genome with variable levels of heterozygosity. Our study highlights several strategies using pre and post-processing approaches to generate haploid assemblies with high continuity and completeness. This benchmark will help users to improve haploid assemblies of non-model organisms, and evaluate the quality of their own assemblies.

**Supplementary Information:**

The online version contains supplementary material available at 10.1186/s12859-021-04118-3.

## Background

With the advent of third-generation sequencing, high-quality assemblies are now commonly achieved for all types of organisms. The rise of two main long-read sequencing companies, Pacific Biosciences (PacBio) and Oxford Nanopore Technologies (Nanopore), has prompted an increase in output as well as a decrease in cost, making their technologies more accessible to research teams and more applicable to any genome, including the more challenging ones with large genome sizes and high repetitive content. The primary advantage of long reads over short reads (such as those generated by Illumina sequencing platforms) is their typical length, which is two or three orders of magnitude greater [1]. As a result, long reads facilitate genome assembly into contigs and scaffolds as they can span repetitive regions [2] and resolve haplotypes [3].

However, long reads typically have a much higher error rate than Illumina data, and these errors are mainly insertions and deletions (vs. substitutions for Illumina reads). PacBio data have a random error pattern that can be compensated with high coverage, and recent developments have aimed to increase accuracy by generating circular consensus sequences [4], where one DNA fragment is read multiple times. Nanopore reads, on the other hand, have systematic errors in homopolymeric regions and therefore Nanopore contigs generally require further correction using Illumina or PacBio reads, in a process called “polishing” [5, 6]. Despite this disadvantage, Nanopore reads are currently much longer than PacBio reads, with runs attaining N50s over 100 kilobases (kb) and longest reads spanning over 1 Megabase (Mb) [7, 8].

This progress has prompted the development of programs dedicated to producing* de novo* assemblies from long reads, all of which follow the Overlap Layout Consensus (OLC) paradigm [9]. Briefly, OLC methods start by building an overlap graph (the “O” step), then simplify it and clean it by applying various heuristics (the “L” step), which typically include the removal of transitively inferable overlaps, and finally compute the consensus sequence of each contig (the “C” step). Some long-read assemblers follow strictly this paradigm, such as Flye [10], Ra [11], Raven [12] (a further development of Ra by the same authors), Shasta [13] and wtdbg2 [14]; whereas other assemblers such as Canu [15] and NextDenovo [16] add a preliminary correction step based on an all-versus-all alignment of the reads (see Table 1). At present, most assemblers aim to generate a haploid assembly, in which each region of the genome is represented exactly once. For diploid or polyploid genomes, haploid assemblies include only one version of each heterozygous region and are therefore reduced representations of the actual complexity of the genome. Haplotypes can be reconstructed from a reference collapsed assembly using long reads with tools such as HapCUT2 [17] and WhatsHap [3]. PacBio reads can also be used to generate haplotype-aware* de novo* assemblies with Falcon-Unzip [18]. Nevertheless, haploid assemblies provide a precious resource for genome analysis as they make it possible to compare easily genome structures, gene sets across species, identify orthologs for phylogenomic analysis, and detect variants across individuals.

**Table 1:** Assemblers included in this study

Assembler	Reads correction	Version used	Access
Canu	$\checkmark$	1.9	github.com/marbl/canu
Flye	$\times$	2.5	github.com/fenderglass/Flye
NextDenovo	$\checkmark$	2.2	github.com/Nextomics/NextDenovo
Ra	$\times$	0.2.1	github.com/lbcb-sci/ra
Raven	$\times$	0.0.7	github.com/lbcb-sci/raven
Shasta	$\times$	0.3.0	github.com/chanzuckerberg/shasta
wtdbg2	$\times$	2.5	github.com/ruanjue/wtdbg2

Long-read assemblers were recently benchmarked on real and simulated PacBio and Nanopore bacterial datasets [19], and all assemblers tested proved their efficiency at reconstructing small haploid genomes within 1 h and with a low RAM usage. For eukaryotic genomes, the wtdbg2 publication [14] included an evaluation of Canu, Flye, Ra and wtdbg2 on several model organisms (*Caenorhabditis elegans*, *Drosophila melanogaster*, *Arabidopsis thaliana* and *Homo sapiens*), all of which had low levels of heterozygosity (at most 1% for *A. thaliana*). The assemblies of *Caenorhabditis elegans* and *Homo sapiens* had small variations in assembly size, as these genomes have a low heterozygosity and are properly collapsed. For *Arabidopsis thaliana*, the Canu assembly was oversized (196.5 Mb) compared to the expected size of 144 Mb, suggesting that the higher heterozygosity of this species led to artefactual duplications. By contrast, all the other assemblies were shorter than the expected size, thus they were likely incomplete and it is unclear how well these assemblers can handle divergent haplotypes. This comparison suggested that wtdbg2 produces fewer artefactual duplications, but no attempt was made to pre-filter reads or post-process assemblies to improve the result. Hence, a comprehensive evaluation of strategies to generate a structurally correct haploid assembly of a non-model, heterozygous diploid organism is still lacking.

To fill this gap, we present here a quantitative and qualitative assessment of seven long-read assemblers on the relatively small eukaryotic genome of the bdelloid rotifer *Adineta vaga*, for which a draft assembly based on short reads was published some years ago [20]. As with most non-model organisms, *Adineta vaga*’s genome presents a mid-range heterozygosity of ca. 2% with a mix of highly heterozygous and low-heterozygosity regions, making such genome more challenging to assemble than those of model organisms, which often exhibit very low levels of polymorphism [21].

In addition to assessing the ability of these seven assemblers to collapse highly heterozygous regions, we investigated whether adding a pre-assembly read-filtering step (selecting reads based on their length and quality) or removing uncollapsed haplotypes post-assembly (using existing tools HaploMerger2 [22], purge_dups [23] and purge_haplotigs [24]) improved the assembly. HaploMerger2 detects uncollapsed haplotypes in assemblies based on sequence similarity alone and can process both low and high-heterozygosity genomes. Along with sequence similarity, purge_dups and purge_haplotigs take into account the coverage depth obtained by mapping short or long reads to the contigs. Coverage depth represents the number of reads covering a position in a contig (computed after mapping reads on the assembly). The contigs are then aligned to select duplicates accurately and remove them. While purge_dups sets its coverage thresholds automatically, purge_haplotigs requires user-provided values. As the focus of the present benchmark was on dealing with uncollapsed haplotypes and not on polishing assemblies (a step for which many tools are available and that would represent a benchmark topic in itself), we did not perform polishing of our contigs.

Assemblies were evaluated using several metrics quantifying their level of continuity and the correctness of their haploid representation of a diploid genome: namely their assembly size, N50, BUSCO completeness, *k*-mer completeness, and coverage distribution. The assembly size represents the sum of the lengths of all the contigs in the assemblies. The N50 is a popular metrics that reflects directly the continuity of the assembly but does not account for possible structural errors; it is defined as the length of the largest contig for which 50% of the assembly size is contained in contigs of equal or greater length. The BUSCO completeness [25] assesses the number of orthologs retrieved completely from the assembly in one or several copies: a high-quality, properly collapsed haploid assembly should exhibit a high number of complete single-copy BUSCO features and a low number of duplicated BUSCO features. The *k*-mer completeness is the percentage of solid (i.e., frequently observed and therefore probably correct) *k*-mers in the set of reads present in the assembly. In the case of a haploid assembly of a diploid genome, all homozygous *k*-mers (i.e., *k*-mers that are shared by the two haplotypes) should be represented in the assembly, whereas only half of the heterozygous *k*-mers (i.e., *k*-mers that are found in only one haplotype) should be represented. To detect both underpurging and overpurging, we focused in our benchmark on the *k*-mer completeness of heterozygous *k*-mers: as we expect only half of them to be present in a haploid assembly, a well-collapsed assembly should exhibit a *k*-mer completeness of about 50%, whereas a lower value indicates that too many *k*-mers were lost (overpurging) and a higher value indicates that too many *k*-mers were retained (underpurging).

We also investigated the coverage-depth distribution of each assembly. In an ideal haploid assembly, all positions should be equally covered, hence we would expect a single peak in the coverage distribution. Based on our analysis of the coverage distribution, we developed a new metric to evaluate the haploidy, or proper collapsing, of assemblies of diploid genomes. The haploidy score is based on the identification of two peaks in the per-base coverage depth distribution: a high-coverage peak that corresponds to bases in collapsed haplotypes (hereafter called “collapsed peak”), and a peak at about half-coverage of the latter that corresponds to bases in uncollapsed haplotypes (“uncollapsed peak”). The haploidy score represents the fraction of collapsed bases in the assembly, and is equal to C/(C+U/2), i.e. the ratio of the area of the collapsed peak (C) divided by the sum of the area of the collapsed peak (C) and half of the area of the uncollapsed peak (U/2). This metric reaches its maximum of 1.0 when there is no uncollapsed peak, in a perfectly collapsed assembly, whereas it returns 0.0 when the assembly is not collapsed at all (as in the case of a phased diploid assembly). We implemented this metric in a new tool called HapPy [26] available at github.com/AntoineHo/HapPy.

Finally, as computational resources can be a limiting factor in genome assembly, we compared the CPU time and RAM usage for the different assemblers tested by running them on the same machine under the same conditions. Canu and NextDenovo were not included in this comparison, as they required significantly higher resources and had to be run on different machines.

## Results

### Preliminary observations

The genome size of our benchmark organism was estimated as 102 Mb based on *k*-mer frequencies (see Methods). The *k*-mer spectrum shows two peaks (Additional file 1: Figure S1): the first one, around 45X, for heterozygous *k*-mers (found in only one haplotype); and the second peak, around 90X, for homozygous *k*-mers (identical in both haplotypes). As *Adineta vaga* has a mid-range heterozygosity, the number of distinct heterozygous *k*-mers is higher than the number of homozygous *k*-mers, making a haploid assembly more difficult to obtain than with low-heterozygosity genomes.

We initially ran each assembler (Table 1) five times on our complete and filtered Nanopore and PacBio datasets, as we had observed that there were some discrepancies (assembly size, N50) in the outputs when running several times Flye, NextDenovo, Shasta and wtdbg2. We found that the assembly size, N50 and BUSCO scores of the resulting assemblies were very similar to each other (Additional file 1: Figures S2–S3). As a result, we chose randomly one replicate assembly from each assembler and used this replicate for the subsequent haplotype-purging step.

To represent assembly statistics in a comprehensive manner, we defined four scores (see Methods): size, that is 1 minus the distance of the assembly size to the expected genome size; N50; completeness, a combined metric that includes both the single-copy BUSCO score and the distance of the observed *k*-mer completeness to the expected value of 50%; haploidy, computed using HapPy.

### Assemblies of PacBio reads

**Fig. 1:**
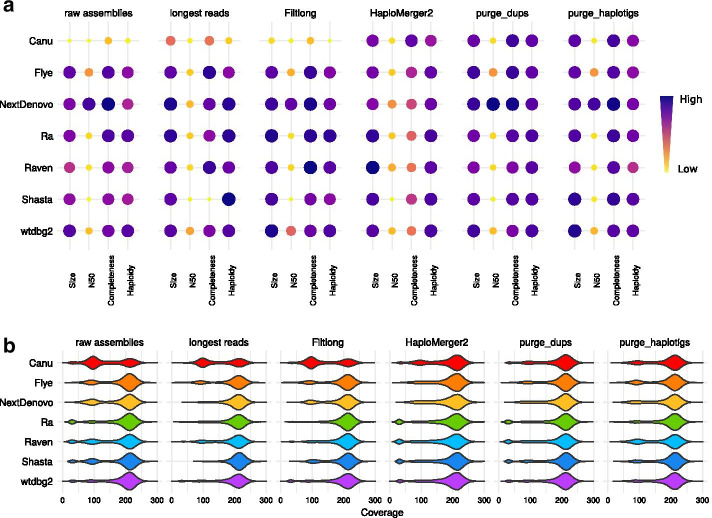
Statistics of PacBio assemblies. Statistics of raw assemblies obtained from the full PacBio dataset (raw assemblies), with a preliminary read-filtering step (keeping only reads larger than 15 kb, or those selected by Filtlong based on quality and length), or a subsequent removal of uncollapsed haplotypes with HaploMerger2, purge_dups, or purge_haplotigs. **a** Assembly scores for size, N50, completeness and haploidy. **b** Long-read coverage distribution over the contigs

Full assembly statistics are available in Additional file 1: Figure S4 and Additional file 1: Table S1–S3, whereas a summary of the results is presented in Fig. 1.

#### Raw assemblies

All raw assemblies of the full PacBio datasets were oversized compared to the estimated genome size of 102 Mb, ranging from 114 Mb (wtdbg2) to 169 Mb (Canu) (Fig. 1a, raw assemblies). The NextDenovo assembly obtained the highest N50 score with a value of 8.9 Mb, while other assemblies had an N50 ranging from 301 kb (Canu) to 2.7 Mb (Flye). The Canu assembly had the lowest completeness score, as its *k*-mer completeness greatly exceeded the expected value of 50% (73.5%) and its number of single-copy BUSCOs was only 538, compared to a highest value of 687 features (NextDenovo). The wtdbg2 and Ra assemblies scored the highest according to the haploidy metrics (0.90), while Canu obtained the lowest score (0.59).

Larger assembly sizes correlated with highly bimodal coverage distributions (Fig. 1b, raw assemblies). This was particularly the case with Canu assemblies, which exhibited two large peaks plus a smaller, low-coverage peak. The high-coverage peak, around 210X, was the collapsed peak C whereas the 100X peak, at about half-coverage of the C peak, corresponded to the uncollapsed peak U. In the case of the Canu assembly, the U peak was larger than the C peak, revealing a poor collapsing of highly heterozygous regions. The Flye, NextDenovo, Raven and Shasta assemblies also exhibited two peaks in their coverage distribution, although their U peak was smaller than the one of Canu. The Ra, Raven, Shasta, and wtdbg2 assemblies exhibited an additional low-coverage peak identified as contaminants (see Additional file 1: Figures S5–S11).

#### Read filtering

We filtered PacBio reads using two strategies: either keeping reads longer than 15 kb; or filtering reads with Filtlong [27] based on quality (in priority) and length. These filtered datasets resulted in assemblies closer to the expected size than assemblies of all reads for Canu, NextDenovo, Ra, Raven and Shasta (Fig. 1a, read filtering). In the case of NextDenovo, filtering based on length made the N50 assembly drop to a value comparable with other assemblies (from 8.9 to 1.8 Mb), while the N50 did not decrease for the Filtlong dataset. Most assemblies maintained their completeness score with both strategies, and it even increased for some (Canu, Flye, Raven). As for the coverage distribution (Fig. 1b, read filtering), the Ra assembly no longer showed a low-coverage contaminant peak. For the NextDenovo assembly the U peak was absent when selecting the longest reads, but remained with the Filtlong dataset. The contaminant peak was also removed for the Raven assembly, and the U peak was reduced. The Shasta assembly had no U peak with the longest reads, but the assembly was shorter than expected (89 Mb) and had a poor completeness score.

#### Haplotig purging

When adding a post-assembly haplotig-purging step, we observed strikingly different results depending on the combination of assembler and post-assembly purging tool, namely HaploMerger2, purge_dups, purge_haplotigs. While HaploMerger2 reduced the size of all assemblies (resulting in higher size scores on Fig. 1a), it also led to a decrease of the completeness score of all assemblies, except for Canu (Fig. 1a, HaploMerger2). Nevertheless, the haploidy scores all increased (with a minimum of 0.84 for Canu and a maximum of 0.92 for Ra and wtdbg2) as U peaks decreased drastically in all coverage distributions (Fig. 1b, HaploMerger2). Assemblies purged with purge_dups were all closer to the expected size of 102 Mb, and the N50 and completeness scores were maintained or even improved (Fig. 1a, purge_dups). The haploidy scores were also improved as they ranged from 0.89 (Canu and Flye) to 0.91 (Ra and wtdbg2). The coverage distributions showed that the U peaks were removed or at least reduced, but the low-coverage peaks were not (Fig. 1b, purge_dups). After purging with purge_haplotigs, all assembly sizes were closer to the expected size except for Flye, and the N50 and completeness scores were maintained or even improved (Fig. 1a, purge_haplotigs). The haploidy scores were improved for Canu, NextDenovo and Shasta. The coverage distributions showed a reduction of the U peak for the Canu, NextDenovo and Shasta assemblies, explaining their higher haploidy scores (Fig. 1b, purge_haplotigs). The low-coverage peaks were removed for Ra, Raven, Shasta and wtdbg2, explaining their smaller assembly size.

#### Combination of read filtering and haplotig purging

The combination of read filtering and haplotig purging resulted in assemblies that almost all had a unimodal coverage distribution (Additional file 1: Figure S12–S13). As observed with assemblies of all reads, HaploMerger2 seemed to overpurge assemblies of the filtered datasets. Only the Canu assembly remained above the expected size, but its statistics were similar to those obtained with the Canu assembly of all reads. Assemblies purged with purge_dups or purge_haplotigs were closer to the estimated size and exhibited high numbers of single-copy BUSCO features. The combination of pre-assembly read filtering and post-assembly purge_dups seemed beneficial for most assemblers with the exception of Shasta, as the resulting assemblies ranged in haploidy from 0.90 (Canu and Flye) to 0.97 (NextDenovo). By contrast, the improvements observed when using a combination of read filtering and purge_haplotigs were similar to those obtained with either one of the two. NextDenovo, Ra and wtdbg2 assemblies had satisfying assembly sizes (from 99 to 107 Mb), high haploidy scores (from 0.85 to 0.96) (Additional file 1: Table S2) and unimodal coverage distributions, but similar scores were also obtained using only read selection for NextDenovo and Ra and using only purge_haplotigs for wtdbg2.

#### Combination of haplotig-purging tools

The combination of purge_dups or purge_haplotigs with HaploMerger2 resulted in problems similar to those observed on assemblies purged only with HaploMerger2: except for Canu, assemblies were shorter than expected and the number of single-copy BUSCO features dropped (Additional file 1: Figure S14). Assemblies purged with both purge_dups and purge_haplotigs were all reduced in size, but none went below the expected size. The BUSCO score and the *k*-mer completeness were stable, and the haploidy scores ranged from 0.88 (Canu and Raven) to 0.92 (NextDenovo) (Additional file 1: Table S3). The coverage distributions were all close to a unimodal distribution, as there were no low-coverage contigs and the U peaks had mostly disappeared.

### Assemblies of Nanopore reads

Full assembly statistics are provided in Additional file 1: Figure S15 and Additional file 1: Table S4–S6.

#### Raw assemblies

**Fig. 2:**
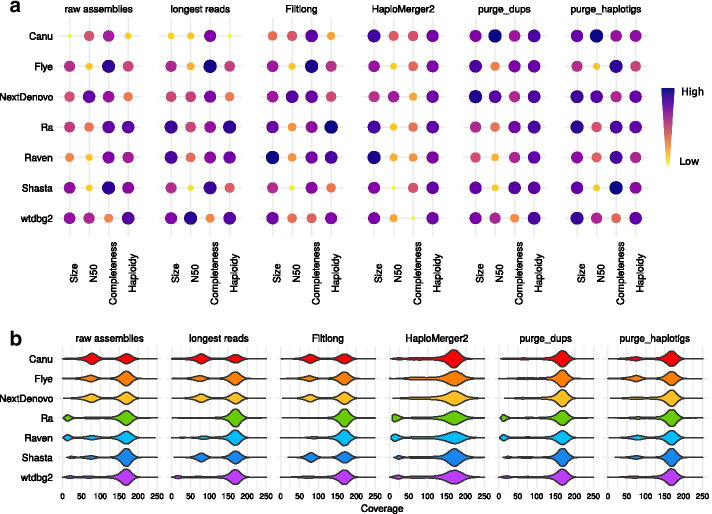
Statistics of Nanopore assemblies. Statistics of raw assemblies obtained from the full Nanopore dataset (raw assemblies), with a preliminary read filtering step (keeping only reads larger than 30 kb, or those selected by Filtlong based on quality and length) or a subsequent removal of uncollapsed haplotypes with HaploMerger2, purge_dups, or purge_haplotigs. **a** Assembly scores for size, N50, completeness and haploidy. **b** Long-read coverage distribution over the contigs

Similarly to the PacBio assemblies, Nanopore assembly sizes exceeded the expected size of 102 Mb, ranging from 118 Mb (Shasta, wtdbg2) to 154 Mb (Canu) (Fig. 2a, raw assemblies). Nanopore assemblies achieved a much higher continuity than PacBio assemblies, as PacBio assemblies had a lowest N50 of 301 kb while Nanopore assemblies had a lowest N50 of 1.6 Mb. Canu, NextDenovo, Ra and wtdbg2 achieved a N50 over 5 Mb using Nanopore reads. The number of complete single-copy BUSCOs was lower in Nanopore assemblies (up to 559) than in PacBio assemblies (up to 699). The *k*-mer completeness was also usually lower for Nanopore assemblies, around 38.6–54.8%, compared to 47.7–73.5% for PacBio assemblies. These lower values for *k*-mer completeness in Nanopore assemblies were likely not due to a better collapsing but rather to systematic errors. The haploidy scores were higher for Nanopore assemblies produced by Canu, Ra, Raven, Shasta and wtdbg2, in comparison with PacBio assemblies, but this score was lower for Flye and NextDenovo assemblies (Additional file 1: Table S1, Additional file 1: Table S4). The coverage distribution of the Canu assembly exhibited two distinct U (uncollapsed) and C (collapsed) peaks respectively around 75X and 160X, indicating that many haplotypes were not collapsed and were therefore represented twice in the assemblies (Fig. 2b, raw assemblies). This was also the case for the Flye, NextDenovo, Raven and Shasta assemblies, albeit their U peak was smaller than the one of the Canu assembly. The Ra, Raven, Shasta and wtdbg2 assemblies had an additional low-coverage peak identified as contaminants (see Additional file 1: Figures S16–S22).

#### Read filtering

When assembling a subset of either the longest Nanopore reads (over 30 kb) or reads filtered with Filtlong, the Ra and Raven assemblies had sizes closer to the estimated size and did not exhibit a contaminant peak in their coverage distribution, whereas other assemblies were generally unmodified (Fig. 2, read filtering). The Raven assembly of filtered reads had a smaller U peak compared to the raw assembly, but still present, whereas the U peak of the Ra assemby disappeared. The wtdbg2 assembly of the Fitlong dataset no longer shows a contaminant peak. These improvements came along with increased haploidy scores: from 0.90 to 0.95–0.97 for Ra, and from 0.83 to 0.89–0.92 for Raven. The result produced by Shasta with read filtering was the opposite, as the size and haploidy scores became lower and the U peak became larger with both filtering strategies. This increase in size of the U peak explained the higher *k*-mer completeness obtained due to a higher percentage of uncollapsed homozygous and heterozygous *k*-mers (Additional file 1: Figure S23–S24). Overall, read filtering did not affect completeness scores.

#### Haplotig purging

As we observed with PacBio reads, all assembly sizes were reduced by HaploMerger2, which resulted in higher size scores, and the U peaks were removed from the coverage distributions, but this also led to lower scores for completeness (Fig. 2, HaploMerger2). purge_dups improved size scores while maintaining or increasing completeness scores and removing U peaks (Fig. 2, purge_dups). These improved coverage distributions resulted in higher haploidy scores, ranging from 0.90 (Flye and Raven) to 0.93 (Ra and Raven). purge_haplotigs improved size scores for all assemblies except Flye and also kept high completeness scores (Fig. 2). Haploidy scores were higher for assemblies produced by Canu and NextDenovo, and the coverage distribution shows that U peak were indeed reduced or removed for Canu and NextDenovo assemblies, while the contaminant peaks were removed for Ra, Raven, Shasta and wtdbg2 assemblies. We observed again that HaploMerger2 generally decreased the continuity and quality metrics of the assemblies, while purge_dups and purge_haplotigs did not. Interestingly, the Canu assembly obtained the highest N50 of all the assemblies presented in this paper (12.4 Mb) when raw assemblies were purged with either purge_dups or purge_haplotigs.

#### Combination of read filtering and haplotig purging

The read-filtered Nanopore assemblies after HaploMerger2 were too short, except for the Canu assembly (Additional file 1: Figure S25–S26). All assemblies of the longest reads after purge_dups were close to the expected genome size (96–109 Mb), except for the wtdbg2 assembly (114 Mb), and the coverage distributions were almost all unimodal, with a haploidy score ranging from 0.87 (Canu) to 0.96 (Ra) (Additional file 1: Table S5). Notably, the Shasta assembly of filtered reads had a strong U peak that was completely removed by purge_dups, which brought the assembly to a size close to our estimation (102 Mb) and resulted in a high number of singe-copy BUSCO features (519 features). purge_haplotigs was apparently less efficient, as the U peak was reduced for the Canu and Raven assemblies but remained high for the Flye, NextDenovo and Shasta assemblies. The BUSCO scores of assemblies purged with purge_dups or purge_haplotigs were similarly high (up to 539 single-copy BUSCO features, Ra).

#### Combination of haplotig-purging tools

The combination of HaploMerger2 with either purge_dups or purge_haplotigs led to excessively shorter assemblies, except for Canu, as HaploMerger2 tended to overpurge (Additional file 1: Figure S27). The assemblies purged with both purge_dups and purge_haplotigs were all close to the expected size (100–110 Mb), their single-copy BUSCO score was maintained (up to 559 features, Flye assembly), they had a *k*-mer completeness below 50%, their haploidy ranged from 0.90 (Flye and Raven) to 0.94 (NextDenovo) (Additional file 1: Table S6) and their coverage distribution was unimodal or close to it.

### Impact of sequencing depth

**Fig. 3:**
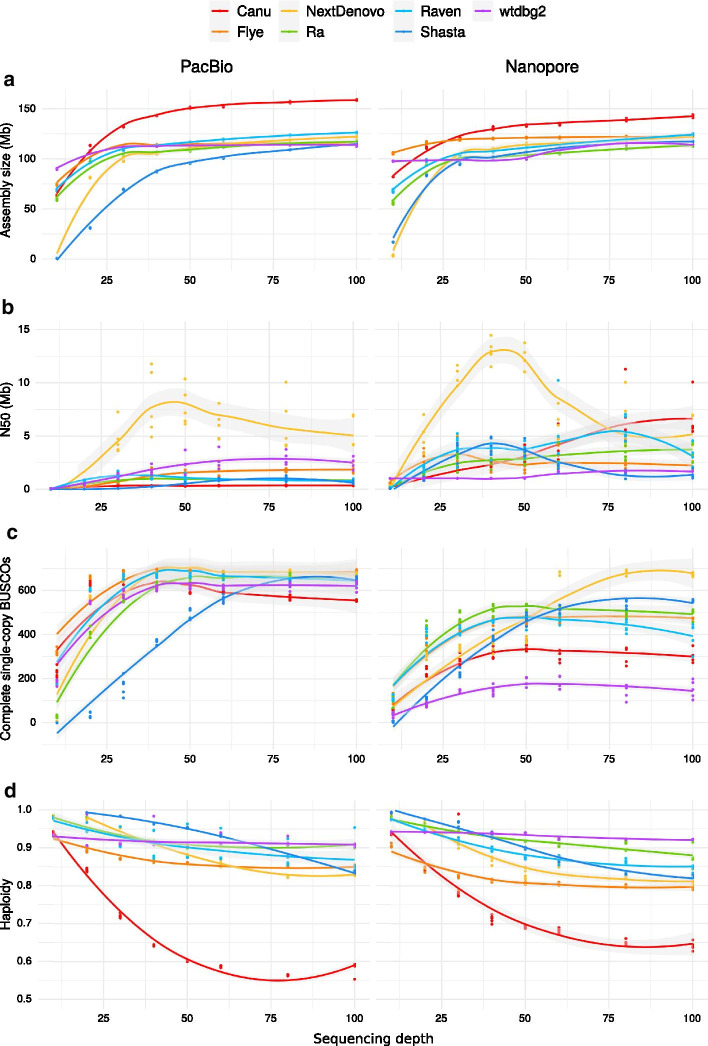
Impact of sequencing depth. Statistics of the PacBio and Nanopore assemblies depending on sequencing depth, with **a** assembly size, **b** N50, **c** complete single-copy BUSCOs and **d** haploidy. The assemblies were ran on five random subsamplings of the long-read datasets

We further evaluated the impact of sequencing depths ranging from 10X to 100X on the size, N50, BUSCO score, and haploidy metrics of PacBio and Nanopore assemblies (Fig. 3).

With a 10X sequencing depth, almost all assemblers produced outputs excessively small compared to the expected genome size, and NextDenovo and Shasta performed the worst. However, with wtbdg2 on PacBio and Nanopore reads, and with Flye on PacBio reads, the assembly size was close to the expected size. At 20X, Canu, Flye, Ra, Raven and wtdbg2 reached at least 93 Mb. Assembly sizes increased sharply up to 40X, except for Canu for which the assemblies kept increasing in size with sequencing depth.

While there was no variation in assembly size among replicates, N50s were highly variable for PacBio assemblies with NextDenovo, Flye and wtdbg2, and for almost all Nanopore assemblies other than wtdbg2. For NextDenovo, there was a clear optimal coverage at about 40X in terms of N50 with PacBio as well as Nanopore reads. Other assemblers also showed a decrease in N50 above a certain sequencing depth: for PacBio assemblies, this happened for Raven over 30X and for Shasta and wtbdg2 over 80X; for Nanopore assemblies, this happened for Flye over 30X, for Shasta over 40X, and for Raven over 80X.

The number of single-copy BUSCO features did not vary much among replicate subsamplings; only NextDenovo exhibited some variability in that regard. Over 40X, the number of single-copy BUSCOs became strikingly stable for most assemblers with both PacBio and Nanopore reads, except for Shasta that is tuned for a 60X depth. For Canu assemblies, the number of complete single-copy BUSCOs decreased when sequencing depth exceeded 30X. For wtdbg2 the complete single-copy BUSCOs remained low for the Nanopore dataset at the different sequencing depths.

For most assemblers, haploidy decreased when sequencing depth increased, and this was especially drastic for Canu assemblies. Only the assemblies produced by wtdbg2 had a stable haploidy value, whereas the Ra assemblies of PacBio reads also exhibited limited variations in haploidy.

### Computational performance

**Fig. 4:**
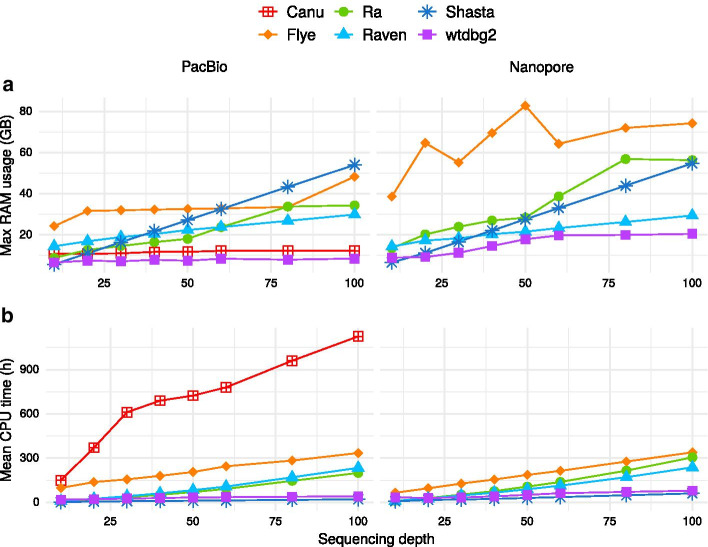
Computational performance. Computational resources (RAM and CPU time) used by the assemblers. **a** Maximum RAM usage and **b** mean CPU time depending on sequencing depth. Canu (for Nanopore reads) and NextDenovo were not included in this comparison as they were run on different machines

We evaluated the computational performance of Flye, Ra, Raven, Shasta and wtdbg2. Canu was only evaluated on PacBio reads as the CPU time was too high for Nanopore reads, and we had to run Canu on a cluster. For the full Nanopore dataset, with 32 threads, Canu ran in 7 days 4 h 41 min and used 99 GB. NextDenovo was not included in this comparison as it required high RAM resources and was therefore run on a different machine; we were not able to measure properly its RAM usage or CPU time. RAM usage and CPU time (Fig. 4) increased with sequencing depth, except for wtdbg2 and Canu that did not display much change. wtdbg2 was the assembler that required the lowest amount of RAM (less than 20 GB) and was the second fastest on PacBio reads (less than 53 h) and Nanopore reads (less than 192 h). Flye had the highest RAM usage for Nanopore assemblies (Canu and NextDenovo excluded), with high variability. Notably, Shasta generally required a lot of RAM and had the highest memory footprint for high-coverage PacBio data, but it ran the fastest on PacBio as well as on Nanopore reads. The most recent versions of Flye (2.8) and Raven (1.5) have decreased the CPU time. Raven was already an efficient assembler, and its latest improvements have halved the RAM usage and CPU time, when tested on the full PacBio and Nanopore datasets. Shasta 0.7.0 also decreased its RAM usage and CPU time.

## Discussion

### Comparison of PacBio and Nanopore assemblies

While PacBio assemblies were superior in terms of completeness, the continuity of Nanopore assemblies was far greater for most assemblers, probably due to the greater length of Nanopore reads. The lower completeness scores of Nanopore assemblies likely resulted from the lower accuracy along with the non-random error pattern of Nanopore reads producing errors (mostly indels) in the consensus sequences produced by the assemblers. These systematic errors in Nanopore reads may be improved with more recent protocols [28] and basecallers [29].

However, there was no striking difference regarding the efficiency of haplotype collapsing when assembling PacBio or Nanopore reads. The results in terms of coverage distribution and haploidy were similar, and it appears therefore that both technologies can be used to produce properly collapsed assemblies. An important finding was that filtering reads based on length and quality improved in many cases the quality of haploid assemblies, as it led to a decrease in coverage depth and to a lower support for both haplotypes, thereby favoring the collapse of the region. We further observed that read filtering did not lead to a decrease in assembly quality as long as the sequencing depth remained sufficient, and for some assemblers read filtering resulted in an increase in N50 and in completeness.

### Successful combinations for haploid assemblies

We found that Canu poorly collapsed alleles and yielded oversized assemblies. The program did not seem able to collapse highly divergent regions on its own. Post-processing assemblies using haplotype-purging tools greatly improved haploidy. All three tools tested reduced the assembly size and the size of the U peak, but purging was most efficient using purge_dups. Purging Canu assemblies with purge_dups or purge_haplotigs improved greatly their haploidy. Besides, Canu assemblies of Nanopore reads purged with purge_dups or purge_haplotigs yielded the highest N50s among all our tests. However, the computational resources required to run Canu may be a limiting factor: although the RAM usage can be limited with the parameter maxMemory, this reduced the number of CPUs used and increased the running time.

Flye assemblies exhibited uncollapsed haplotypes too; selecting the longest reads did not help, but purge_dups improved collapsing, brought the assembly size close to the expectation and kept a good quality.

NextDenovo produced the assemblies with the highest N50s before post-processing, but with poorly collapsed haplotypes. This problem was alleviated for PacBio assemblies when selecting the longest reads, and uncollapsed haplotypes were efficiently removed by haplotig-purging tools. The best haploidy values were achieved when combining NextDenovo with read filtering, purge_dups and purge_haplotigs. These assemblies also reached high values of continuity and completeness. However, although NextDenovo runs quickly, it requires a large amount of RAM.

Ra was more efficient at collapsing haplotypes than most assemblers, and its oversized assemblies were rather due to contaminants. Ra assemblies proved even better when using only the longest reads, which led to a better continuity, equal completeness and improved collapsing. Although Ra was not the most computationally efficient assembler, its RAM usage and CPU time remained low up to a sequencing depth of 50X; thus it appeared even more desirable to use only a subset of the longest reads for this assembler.

Raven is a further development of Ra, yet it exhibited a different behavior: Raven was more computationally efficient but produced less collapsed haplotypes compared to Ra. Read filtering and haplotig-purging reduced these uncollapsed haplotypes, resulting in high-quality assemblies when combining read filtering with purge_dups, or with purge_dups and purge_haplotigs. The low RAM usage and runtime of the newest version make it a compelling assembler.

We observed singular results with Shasta. The assembly of filtered Nanopore reads was less collapsed than the assembly of all reads. purge_dups efficiently purged the assemblies of all PacBio and Nanopore reads but, surprisingly, the best haploid Shasta assembly was obtained from filtered Nanopore reads purged with purge_dups. Shasta assemblies generally achieved a good completeness, but their continuity was lower than with other programs, as the developers explicitly aimed for quality over continuity.

wtdbg2 performed well on PacBio data, but less on Nanopore reads, for which it obtained the lowest completeness scores. This program did not seem to have difficulties with heterozygous regions, but low-coverage contigs identified as contaminants remained in the final assemblies. Read selection on size did not significantly improve the assemblies, but purge_haplotigs removed contaminant contigs, therefore improving the output. Short-read polishing would certainly improve the low completeness of Nanopore assemblies. Users may want to test this assembler as it collapses genomes well and runs fast using a moderate amount of RAM.

Based on the above, we recommend users interested in generating the best haploid assembly of a diploid genome to try all or some of the solutions described in Table 2, depending on the size of the genome they want to assemble, the technology of reads they have, and their available computational resources.

**Table 2:** Recommended strategies for generating high-quality haploid assemblies

Assemblers	Recommended strategies	Advantages
Canu	purge_dups and/or purge_haplotigs	Highest N50 (Nanopore)
Flye	purge_dups	
NextDenovo	read filtering, purge_dups and/or purge_haplotigs	High continuity
Ra	read filtering	Low RAM usage and CPU time
Raven	read filtering + purge_dups or purge_dups + purge_haplotigs	Low RAM usage and CPU time
Shasta	purge_dups	Low CPU time
wtdbg2	purge_haplotigs	Lowest RAM usage and CPU time

### Impact of sequencing depth

Our study of the impact of sequencing depth on the assemblies showed that deeper sequencing usually did not result in higher continuity or improved haploidy of the assemblies. Most programs reached the expected assembly size between 10X and 30X, while the BUSCO scores and *k*-mer completeness plateaued around 40X. Depending on the assembler, N50s also decreased when sequencing depth went beyond a specific threshold. A deeper coverage may lead to erroneous low-coverage contigs and provide more support to both haplotypes in highly heterozygous regions, promoting incomplete collapsing of haplotypes. We observed in our benchmark that a deeper sequencing led to a lower haploidy (as computed using HapPy). The combination of the continuity and quality metrics show that a sequencing depth of 40X is sufficient for generating a high-quality haploid assembly. Besides, a counter-intuitive finding is that a larger amount of reads does not improve the assembly and can even make it worse in terms of continuity and haploidy. Most assemblers seem optimized for sequencing depths around 30 to 40X and therefore did not appear to benefit from more data, except Shasta that is optimized for 60X.

### Assembly evaluation

We propose here a set of metrics to evaluate thoroughly genome assemblies and identify uncollapsed haplotypes. The N50, BUSCO score and *k*-mer completeness are commonly used to estimate the continuity and completeness of assemblies, but do not discriminate properly collapsed haploid assemblies. It is possible to combine the completeness and continuity with a comparison of assembly size vs. expected genome size and an examination of the coverage distribution to identify the best assemblies. We further described a new metric of the haploidy of an assembly and implemented it in HapPy. We used the haploidy metric to systematically evaluate haploid assemblies. HapPy gives an accurate numerical representation of the coverage distribution.

These metrics have their limits, and not one of them is sufficient to identify the best assembly. The N50 is the most popular metric to describe contigs as it represents the continuity, yet high N50s can be achieved with efficient scaffolding methods. Therefore, we should aim for high-quality contigs that can be later turned into high-quality scaffolds. Comparing the assembly size to an estimated genome size depends on the reliability of the estimation itself. Genome size can be estimated computationally with a *k*-mer spectrum [30, 31], or experimentally using flow cytometry and Feulgen densitometry [32]. The BUSCO score only represents orthologs and does not account for the completeness of non-coding regions. Besides, the *k*-mer completeness is not sufficient as a value, as it could reach 50% with an assembly that has a balanced combination of missing regions and uncollapsed ones. To better estimate the *k*-mer completeness, it is necessary to examine plots provided by KAT. Collapsed repeated regions appear in the coverage plot along the contigs as localized peaks of elevated coverage. Since most assemblers included in our benchmark can produce an assembly graph, we strongly advise readers to investigate this file using dedicated tools [33]: uncollapsed haplotypes are usually observed as bubbles in the graph. Ideally, the contigs should be evaluated with all metrics available to find, if not the perfect assembly, the best assembly, while assessing its limitations.

Genome papers present only the most successful assembly strategy, but researchers usually try more than one method to obtain the best result. They rarely report details of all the tested approaches in publications, although such negative results could help the community and could guide developers in how to improve their tools. To improve on this situation, we suggest reporting alternative assembly results as additional file or separately on a preprint platform.

### Comparison with other studies

Few comparisons of long-read assemblers are currently available. A thorough study was conducted on simulated and real bacterial datasets [19], and showed that all these assemblers can achieve assemblies of varying quality. Flye and Raven were also tested in this study and emerged among the most reliable assemblers. We also found that these assemblers reached high single-copy BUSCO scores, but when processing data from a diploid organism, they are not the most efficient at collapsing haplotypes. Besides, the benchmark on bacterial datasets also showed that Canu required more computational time and memory than most assemblers. Although this benchmark gave essential information on the performance of these assemblers, eukaryotic genomes represent a completely different challenge. Recently, a publication compared different long-read sequencing technologies to assemble a plant genome, *Macadamia jansenii* [34]. They included statistics for different assemblers and obtained, depending on the tool, oversized assemblies combined with heightened numbers of duplicated BUSCO features on an 80X PacBio dataset, while they did not observe such differences on a 30X Nanopore dataset. These results agree with our observations on the impact of sequencing depth, as the 30X Nanopore dataset was not problematic, while the 80X PacBio dataset was, likely because of a deeper sequencing.

### Toward high-quality diploid and polyploid assemblies

Haploid assemblies of multiploid (i.e. diploid or polyploid) organisms provide a partial representation of their genomes as only one version of all heterozygous regions is included in the assembly. Ideally, we would prefer to generate phased multiploid assemblies. Low-accuracy long reads can separate haplotypes for highly heterozygous regions, but their high error rates do not allow the identification and separation of small heterozygous regions. Furthermore, phased assemblies bring an extra challenge, as alleles from different heterozygous regions need to be correctly associated. A protocol for high-accuracy long reads (above 99%) has been released recently, called PacBio HiFi [4], and brings new possibilities for phased multiploid assemblies. To better accommodate these high-accuracy long reads, new versions of assemblers have been released such as HiCanu [35] (a development of Canu), hifiasm [36], or Flye’s new option --pacbio-hifi. Fully phased assemblies will provide complete representations of multiploid genomes.

## Conclusion

We tested seven long-read assemblers on PacBio and Nanopore for a non-model eukaryote genome. As this genome has variable levels of heterozygosity, including highly heterozygous regions, we found that most assemblers had difficulties collapsing divergent haplotypes, resulting in oversized assemblies. Ra and wtdbg2 emerged as the most efficient programs to obtain haploid assemblies. We identified several assembly strategies combining assemblers, pre-assembly read filtering and post-assembly haplotig purging tools (either purge_dups or purge_haplotigs) that led to properly collapsed haploid assemblies, and also improved continuity and completeness. To guide users when filtering datasets, we tested assemblers with different sequencing depths, and found that aiming for a sequencing depth of 40X could optimize haplotype collapsing and continuity. In addition, we also conducted a thorough evaluation of these assemblies using N50, BUSCO and *k*-mer completeness, coverage distribution, and a new metric of haploidy that we implemented in HapPy. We recommend to reproduce these evaluations for any haploid assembly of a multiploid genome to ensure proper collapsing and avoid artefactual duplications.

We believe that benchmarks such as ours are essential to help researchers working on non-model organisms select a long-read sequencing technology and an assembly method suitable for their project. It will also help them better understand the results they obtain thereby improving the rapidly evolving field of genomics.

## Methods

All command lines are provided in Additional file 1: Table S7.

### Genome size estimation

The genome size of *Adineta vaga* was estimated using KAT v2.4.2 [30] on an Illumina dataset of 25 millions paired-end 250 basepairs (bp) reads (see Additional file 1: Table S2). The diploid size was estimated to 204.6 Mb, therefore a haploid assembly should have a length around 102.3 Mb.

### Long-read assemblies

Canu, Flye, NextDenovo, Ra, Raven, Shasta and wtdbg2 were tested on two *Adineta vaga* long-read datasets: PacBio reads totalling 23.5 Gb with a N50 of 11.6 kb; and Nanopore reads totalling 17.5 Gb with a N50 of 18.8 kb (after trimming using Porechop v0.2.4, github.com/rrwick/Porechop). All assemblers were used with default parameters, except for Shasta for which the minimum read length was set to zero (instead of the default 10 kb setting). To run Shasta on PacBio reads, we used the recommended parameters --Assembly.consensusCaller Modal --Kmers.k 12. When assemblers required an estimated size, the value 100 Mb was provided. PacBio assemblies were run on all reads and on reads > 15 kb (4.7 Gb). Nanopore assemblies were run on all reads and on reads > 30 kb (5.7 Gb). For both datasets, we tested several length thresholds to find the optimal one. For more details on the long-read datasets we used, see the publication by Simion et al. [37] and Additional file 1: Table S8. To test for reproducibility, all assemblers were run five times.

### Read filtering

Reads were filtered following two strategies: keeping only the reads larger than 15 kb (PacBio reads) or 30 kilobases (Nanopore reads); using Filtlong v0.2.0. Filtlong was run with the parameters --target_bases 4092000000 –mean_q_weight 10 to keep about 40X of data and give a priority to quality over length.

### Purging duplicated regions

Reads were mapped on assemblies using minimap2 v2.17-r941 [38]. For each assembly, we ran purge_haplotigs hist [24] to compute coverage histograms that we used to set low, mid and high cutoffs; these values were then used by purge_haplotigs cov to detect suspect contigs. Finally, we ran purge_haplotigs purge to eliminate duplicated regions.

purge_dups [23] was run following instructions by first generating the configuration file and then purging the assembly. HaploMerger2 [22] was run by sequentially running the modules BuildDatabase, RepeatModeler, RepeatMasker and finally the main script of HaploMerger2 to purge the assembly.

### Impact of sequencing depth

To find out the impact of sequencing depth, five replicate subsets were randomly sampled from the long-read datasets using the script reformat.sh from BBTools v38.79, available at sourceforge.net/projects/bbmap/, by providing the desired number of bases. The assemblers were run on these subsets with the same parameters as previously, with the exception of Canu: the parameters stopOnLowCoverage was set to 1 to allow runs on low-depth datasets. We tested subsets with a sequencing depth of 10X, 20X, 30X, 40X, 50X, 60X, 80X, 100X.

### Assembly evaluation

To evaluate the assemblies, we ran BUSCO v4 [25] against metazoa odb10 (954 features) without the parameter --long. We ran KAT comp v 2.4.2 [30] to calculate *k*-mer completeness by reference to the same Illumina 2*250 bp dataset used to estimate the genome size. To compute coverage, long reads were mapped on one replicate assembly per assembler using minimap2 and the coverage was computed with tinycov, available at github.com/cmdoret/tinycov, with a window size of 20 kb.

### Haploidy evaluation

To evaluate the collapsing of assemblies based on the coverage distribution, we developed a script, available at github.com/AntoineHo/HapPy. HapPy estimates the haploidy of an assembly (a measure of how well it is collapsed) by analyzing the per-base coverage histogram obtained after mapping reads to the assembly. For a well-collapsed haploid assembly, this histogram should consist of one peak around the theoretical average depth of coverage.

HapPy takes a raw coverage frequency histogram as input. First, to avoid problems due to local variations and noise in the dataset, two filters are applied before attempting to find peaks in the dataset. The first filter avoids misdetections due to potential peaks at very large coverage. These can be due to repetitive DNA for instance. These peaks are eliminated from the curve by using a filter on the cumulative sum of the frequency of each coverage value. For each increasing coverage value, the total frequency sum is incremented by the frequency of this coverage value. When this sum exceeds 99% of the sum of frequencies of all coverage values, then the remaining coverage values are discarded. Effectively, this discards the very large coverage bins containing low information peaks. The second filter is applied on the resulting histogram curve. It smoothes the curve using a Savitzky-Golay filter [39] from the SciPy package. This filter uses convolution to smooth the curve by fitting a low-degree polynomial function (using linear least squares) to consecutive subsets of adjacent data points. The window length used is 41 and the polynomial degree used is 3.

Then SciPy is used to detect local maxima in the curve. A local maximum is defined as a sample in the input array for which any neighbouring sample has a smaller amplitude. Local peaks detected are filtered by SciPy using different parameters such as the minimum height to be considered a peak and the prominence of a peak compared to its neighboring local maxima. Finally, to determine peak widths, SciPy uses an algorithm described in its documentation: https://docs.scipy.org/doc/scipy/reference/generated/scipy.signal.peak_widths.html#scipy.signal.peak_widths.

Actual coverage curves rarely match the theoretical expectation of a single peak for a haploid assembly because of several factors:

- contaminant contigs (e.g. bacteria and viruses that were sequenced along the organism of interest) can appear as additional peaks at unexpected coverage values (usually lower than the actual sequencing depth);
- some contigs or contig regions in the assembly may actually correspond to uncollapsed haplotigs;
- large and/or abundant hemizygous deletions, as well as haploid chromosomes (e.g. the Y chromosome of male mammals), can result in a half-coverage peak; in such case, this peak is a biological signal that will prevent reaching a perfect haploidy score.

HapPy expects up to three peaks in the per-base coverage histogram: a low-coverage contaminant peak (if any), an uncollapsed peak at around half of the expected coverage and a collapsed peak around the expected coverage.

After peak detection, HapPy determines whether peaks are contaminant, collapsed or uncollapsed based on the thresholds given by the user in input parameters and their relative positions in the curve. Then, it finds the actual limits between contaminants, collapsed and uncollapsed regions of the curve using the computed peak widths. The peak area corresponds to the number of bases in the specific range of coverage values that was attributed to each peak.

We defined a haploidy score using the following Equation 1, in which *U* is the area of the uncollapsed peak and *C* the area of the collapsed peak. It describes the proportion between bases that are collapsed and the total number of bases that we expect in a perfectly haploid assembly. A perfectly haploid assembly is theoretically an assembly of a diploid organism for which all bases from one haplotype have an homologous representation on the other haplotype. For such an assembly, the haploidy score would be equal to 1.0. However, this equation does not take into account insertions and deletions (indels). If indels are numerous or large, then the best scores that the assembly could reach would be lower than 1.0 because all bases do not have an homologous equivalent on the alternative haplotype. For instance, an insertion will not have, by definition, a homologous counterpart; thus this homologous region is not present to be sequenced, which will result in a halved coverage over the insertion region.

1 $$\begin{aligned} Haploidy = \frac{C}{C+\frac{U}{2}} \end{aligned}$$

Note that Eq. 1 does not take into account bases that were attributed to contaminant sequences based on the coverage curve. Ideally, the contigs should be pre-filtered for obvious contaminant taxa before using HapPy.

### Scoring

We defined four scores to evaluate the assemblies in Figs. 1 and 2: size, N50, completeness and haploidy. The N50 score corresponds to the regular N50 value, and the haploidy score is computed using HapPy. The size score reflects the distance of the assembly size to the estimated haploid genome size and is computed following Eq. 2, in which *s* is the assembly size and *G* the estimated haploid genome size (102 Mb).

2 $$\begin{aligned} S = 1 - \frac{Abs\left( s - G\right) }{G} \end{aligned}$$

The completeness score includes both the number of single-copy BUSCO features and a measure of the distance of the observed *k*-mer completeness compared to the expected one. This metrics is computed using Eq. 3, in which $k_{obs}$ is the observed *k*-mer completeness, $k_{exp}$ the expected *k*-mer completeness and $k_{exp}=50$.

3 $$\begin{aligned} K = 1 - Abs\left( k_{obs} - k_{exp}\right) /k_{exp} \end{aligned}$$

The number of single-copy BUSCOs and the value *K* are normalized on a 0 to 1 scale following Eq. 4, in which $x_{i}$ is the initial value, $x_{min}$ the minimum value for all PacBio or Nanopore assemblies, and $x_{max}$ the maximum value for all PacBio or Nanopore assemblies.

4 $$\begin{aligned} x_{f} = \left( x_{i} - x_{min}\right) /\left( x_{max} - x_{min}\right) \end{aligned}$$

The final completeness score is computed using Eq. 5, in which $B_{norm}$ is the normalized single-copy BUSCO score and $K_{norm}$ is the normalized *k*-mer completeness value computed previously.

5 $$\begin{aligned} Comp = \left( B_{norm} + K_{norm}\right) /2 \end{aligned}$$

### Performance evaluation

For Flye, Ra, Raven, Shasta and wtdbg2, maximal RAM usage and mean CPU time were measured using the command time with 14 threads on a computer with an i9-9900X 3.5 Ghz processor and 128 GB RAM. NextDenovo was run on a computer with an Intel Xeon E5-2650 with 256 GB of RAM, while Canu was run on a cluster with an AMD Epyc 7551P and 256 Gb of RAM. NextDenovo ran in a few hours, while Canu runs each required several days.

## Supplementary Information

**Additional file 1**. This file includes all Supplementary Figures S1–27 and Tables S1–S8. 

## Data Availability

The datasets analyzed in this study were published in [37].

## Abbreviations

kb: kilobase
Mb: Megabase
Nanopore: Oxford Nanopore Technologies
OLC: Overlap Layout Consensus
PacBio: Pacific Biosciences

## References

[1] Sedlazeck FJ, Lee H, Darby CA, Schatz MC (2018). Piercing the dark matter: bioinformatics of long-range sequencing and mapping. Nat Rev Genet.

[2] Pollard MO, Gurdasani D, Mentzer AJ, Porter T, Sandhu MS (2018). Long reads: their purpose and place. Hum Mol Genet.

[3] Patterson MD, Marschall T, Pisanti N, Van Iersel L, Stougie L, Klau GW, Schönhuth A (2015). WhatsHap: weighted haplotype assembly for future-generation sequencing reads. J Comput Biol.

[4] Wenger AM, Peluso P, Rowell WJ, Chang P-C, Hall RJ, Concepcion GT, Ebler J, Fungtammasan A, Kolesnikov A, Olson ND, Töpfer A, Alonge M, Mahmoud M, Qian Y, Chin C-S, Phillippy AM, Schatz MC, Myers G, Depristo MA, Ruan J, Marschall T, Sedlazeck FJ, Zook JM, Li H, Koren S, Carroll A, Rank DR, Hunkapiller MW (2019). Accurate circular consensus long-read sequencing improves variant detection and assembly of a human genome. Nat Biotechnol.

[5] Kundu R, Casey J, Sung W-K (2019). HyPo: super fast & accurate polisher for long read assemblies. bioRxiv.

[6] Zimin AV, Salzberg SL (2020). The genome polishing tool POLCA makes fast and accurate corrections in genome assemblies. PLoS Comput Biol.

[7] Jain M, Koren S, Miga KH, Quick J, Rand AC, Sasani TA, Tyson JR, Beggs AD, Dilthey AT, Fiddes IT, Malla S, Marriott H, Nieto T, O’Grady J, Olsen HE, Pedersen BS, Rhie A, Richardson H, Quinlan AR, Snutch TP, Tee L, Paten B, Phillippy AM, Simpson JT, Loman NJ, Loose M (2018). Nanopore sequencing and assembly of a human genome with ultra-long reads. Nat Biotechnol.

[8] Miga KH, Koren S, Rhie A, Vollger MR, Gershman A, Bzikadze A, Brooks S, Howe E, Porubsky D, Logsdon GA, Schneider VA, Potapova T, Wood AM (2019). Jonat: telomere-to-telomere assembly of a complete human X chromosome. bioRxiv.

[9] Miller JR, Koren S, Sutton G (2010). Assembly algorithms for next-generation sequencing data. Genomics.

[10] Kolmogorov M, Yuan J, Lin Y, Pevzner PA (2019). Assembly of long, error-prone reads using repeat graphs. Nat Biotechnol.

[11] Vaser R, Šikić M. Yet another *de novo* genome assembler. In: International symposium on image and signal processing and analysis, ISPA. 2019. p. 147–51. 10.1109/ISPA.2019.8868909.

[12] Vaser R, Šikić M (2020). Raven: a de novo genome assembler for long reads. bioRxiv.

[13] Shafin K, Pesout T, Lorig-roach R, Haukness M, Olsen HE, Bosworth C, Armstrong J, Tigyi K, Maurer N, Koren S, Sedlazeck FJ, Marschall T, Mayes S, Costa V, Zook JM, Liu KJ, Kilburn D, Sorensen M, Munson KM, Vollger MR, Monlong J, Garrison E, Eichler EE, Salama S, Haussler D, Green RE, Akeson M, Phillippy A, Miga KH, Carnevali P, Jain M, Paten B (2020). Nanopore sequencing and the Shasta toolkit enable efficient de novo assembly of eleven human genomes. Nat Biotechnol.

[14] Ruan J, Li H (2020). Fast and accurate long-read assembly with wtdbg2. Nat Methods.

[15] Koren S, Walenz BP, Berlin K, Miller JR, Bergman NH, Phillippy AM (2017). Canu: scalable and accurate long-read assembly via adaptive* k*-mer weighting and repeat separation. Genome Res.

[16] NextOmics: NextDeNovo. 2019. https://github.com/Nextomics/NextDenovo.

[17] Edge P, Bafna V, Bansal V (2017). HapCUT2: robust and accurate haplotype assembly for diverse sequencing technologies. Genome Res.

[18] Chin C-S, Peluso P, Sedlazeck FJ, Nattestad M, Concepcion GT, Clum A, Dunn C, O’Malley R, Figueroa-Balderas R, Morales-Cruz A (2016). Phased diploid genome assembly with single-molecule real-time sequencing. Nat Methods.

[19] Wick RR, Holt KE. Benchmarking of long-read assemblers for prokaryote whole genome sequencing. F1000Research 8, 2019;2138. 10.12688/f1000research.21782.1. 10.12688/f1000research.21782.1 PMC6966772 31984131

[20] Flot JF, Hespeels B, Li X, Noel B, Arkhipova I, Danchin EGJ, Hejnol A, Henrissat B, Koszul R, Aury JM, Barbe V, Barthélémy RM, Bast J, Bazykin GA, Chabrol O, Couloux A, Da Rocha M, Da Silva C, Gladyshev E, Gouret P, Hallatschek O, Hecox-Lea B, Labadie K, Lejeune B, Piskurek O, Poulain J, Rodriguez F, Ryan JF, Vakhrusheva OA, Wajnberg E, Wirth B, Yushenova I, Kellis M, Kondrashov AS, Welch DBM, Pontarotti P, Weissenbach J, Wincker P, Jaillon O, Van Doninck K (2013). Genomic evidence for ameiotic evolution in the bdelloid rotifer Adineta vaga. Nature.

[21] Leffler EM, Bullaughey K, Matute DR, Meyer WK, Ségurel L, Venkat A, Andolfatto P, Przeworski M (2012). Revisiting an old riddle: what determines genetic diversity levels within species?. PLoS Biol.

[22] Huang S, Kang M, Xu A (2017). HaploMerger2: rebuilding both haploid sub-assemblies from high-heterozygosity diploid genome assembly. Bioinformatics.

[23] Guan D, McCarthy SA, Wood J, Howe K, Wang Y, Durbin R (2020). Identifying and removing haplotypic duplication in primary genome assemblies. Bioinformatics.

[24] Roach MJ, Schmidt SA, Borneman AR (2018). Purge haplotigs: allelic contig reassignment for third-gen diploid genome assemblies. BMC Bioinform.

[25] Simão FA, Waterhouse RM, Ioannidis P, Kriventseva EV, Zdobnov EM (2015). BUSCO: assessing genome assembly and annotation completeness with single-copy orthologs. Bioinformatics.

[26] Houtain A, Guiglielmoni N, Flot J-F (2020). AntoineHo/HapPy: v0.1 (version v0.1zen). Zenod.

[27] Wick RR. Filtlong. 2017. https://github.com/rrwick/Filtlong.

[28] Van der Verren SE, Van Gerven N, Jonckheere W, Hambley R, Singh P, Kilgour J, Jordan M, Wallace EJ, Jayasinghe L, Remaut H (2020). A dual-constriction biological nanopore resolves homonucleotide sequences with high fidelity. Nat. Biotechnol..

[29] Wick RR, Judd LM, Holt KE (2019). Performance of neural network basecalling tools for Oxford Nanopore sequencing. Genome Biol.

[30] Mapleson D, Garcia Accinelli G, Kettleborough G, Wright J, Clavijo BJ (2016). KAT: a K-mer analysis toolkit to quality control NGS datasets and genome assemblies. Bioinformatics.

[31] Ranallo-Benavidez TR, Jaron KS, Schatz MC (2020). GenomeScope 2.0 and Smudgeplot for reference-free profiling of polyploid genomes. Nat Commun.

[32] Mulligan KL, Hiebert TC, Jeffery NW, Gregory TR (2014). First estimates of genome size in ribbon worms (phylum Nemertea) using flow cytometry and Feulgen image analysis densitometry. Can J Zool.

[33] Wick RR, Schultz MB, Zobel J, Holt KE (2015). Bandage: interactive visualization of de novo genome assemblies. Bioinformatics.

[34] Murigneux V, Rai SK, Furtado A, Bruxner TJ, Tian W, Harliwong I, Wei H, Yang B, Ye Q, Anderson E (2020). Comparison of long-read methods for sequencing and assembly of a plant genome. GigaScience.

[35] Nurk S, Walenz BP, Rhie A, Vollger MR, Logsdon GA, Grothe R, Miga KH, Eichler EE, Phillippy AM, Koren S (2020). HiCanu: accurate assembly of segmental duplications, satellites, and allelic variants from high-fidelity long reads. Genome Res.

[36] Cheng H, Concepcion GT, Feng X, Zhang H, Li H. Haplotype-resolved *de novo* assembly with phased assembly graphs. Nat. Methods. 2021;18(2), 170-5. 10.1038/s41592-020-01056-5. 10.1038/s41592-020-01056-5 PMC7961889 33526886

[37] Simion P, Narayan J, Houtain A, Derzelle A, Baudry L, Nicolas E, Cariou M, Guiglielmoni N, Kozlowski DKL, Gaudray FR, Terwagne M, Virgo J, Noel B, Wincker P, Danchin EGJ, Marbouty M, Hallet B, Koszul R, Limasset A, Flot J-F, Van Doninck K (2020). Homologous chromosomes in asexual rotifer Adineta vaga suggest automixis. bioRxiv.

[38] Li H (2018). Minimap2: pairwise alignment for nucleotide sequences. Bioinformatics.

[39] Savitzky A, Golay MJ (1964). Smoothing and differentiation of data by simplified least squares procedures. Anal Chem.

