# Supplementary material for: Overcoming uncollapsed haplotypes in long-read assemblies of non-model organisms
Source: BMC Bioinformatics. 2021 Jun 5;22:303. doi: 10.1186/s12859-021-04118-3 (PMC8178825; doi:10.1186/s12859-021-04118-3)
Supplement: Supplementary file 1 — Additional file 1. This file includes all Supplementary Figures S1–27 and Tables S1–S8. [file 12859_2021_4118_MOESM1_ESM.pdf]

Supplementary data

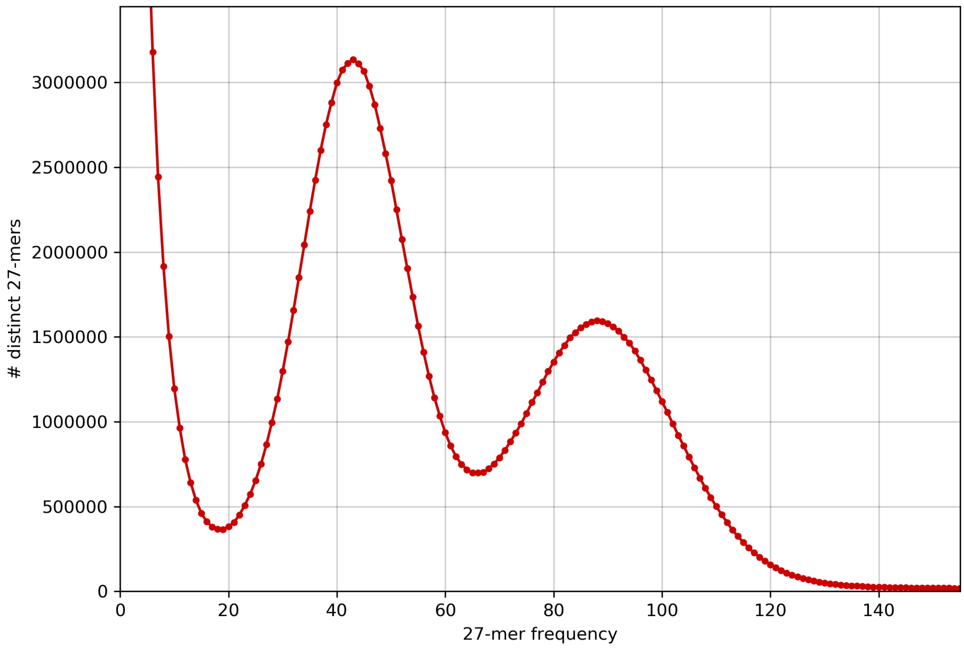

Figure S1. *k*-mer spectrum of *Adineta vaga* using Illumina reads and KAT v2.4.2. The first peak corresponds to heterozygous *k*-mers (around 45X) and the second peak corresponds to homozygous *k*-mers.

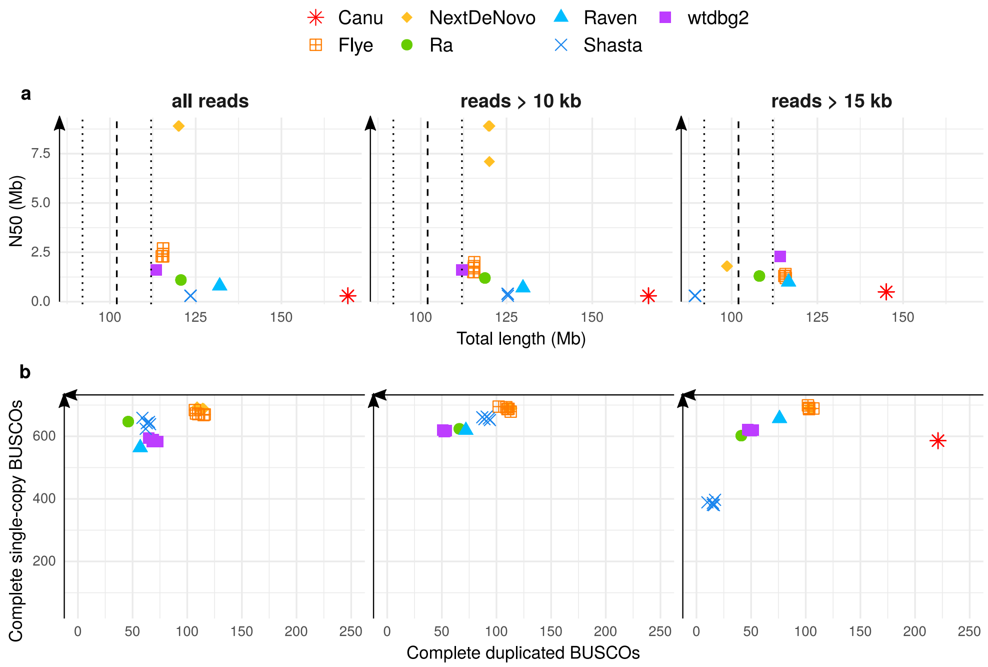

Figure S2. Statistics of PacBio assemblies obtained from the full PacBio dataset or with a read-filtering step prior to assembly based on read length exclusively, using different thresholds: 10 kb, 15 kb. All assemblies were run five times to assess the reproducibility of the output produced by each assembler. a) N50 plotted against total assembly length. The dashed line indicates the expected genome size, with a +/- 10 Mb margin delimited by the dotted lines. b) Number of complete single-copy BUSCOs plotted against number of complete duplicated BUSCOs, from a total of 954 orthologs.

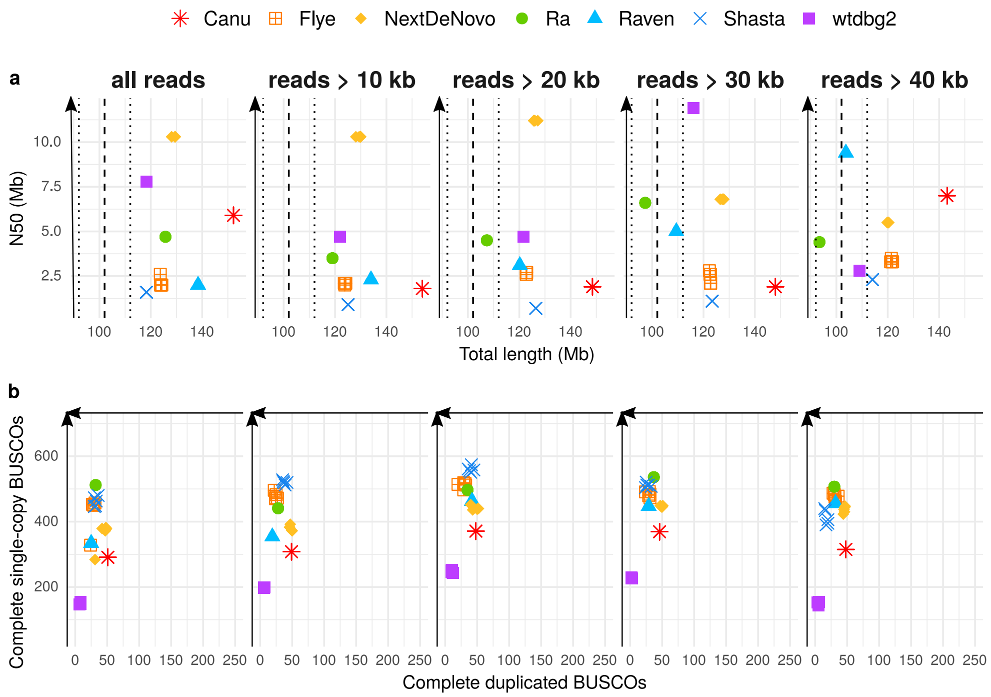

Figure S3. Statistics of Nanopore assemblies obtained from the full Nanopore dataset or with a read-filtering step prior to assembly based on read length exclusively, using different thresholds: 10 kb, 20 kb, 30 kb, 40 kb. All assemblies were run five times to assess the reproducibility of the output produced by each assembler. a) N50 plotted against total assembly length. The dashed line indicates the expected genome size, with +/- 10 Mb margin delimited by the dotted lines. b) Number of complete single-copy BUSCOs plotted against number of complete duplicated BUSCOs, from a total of 954 orthologs.

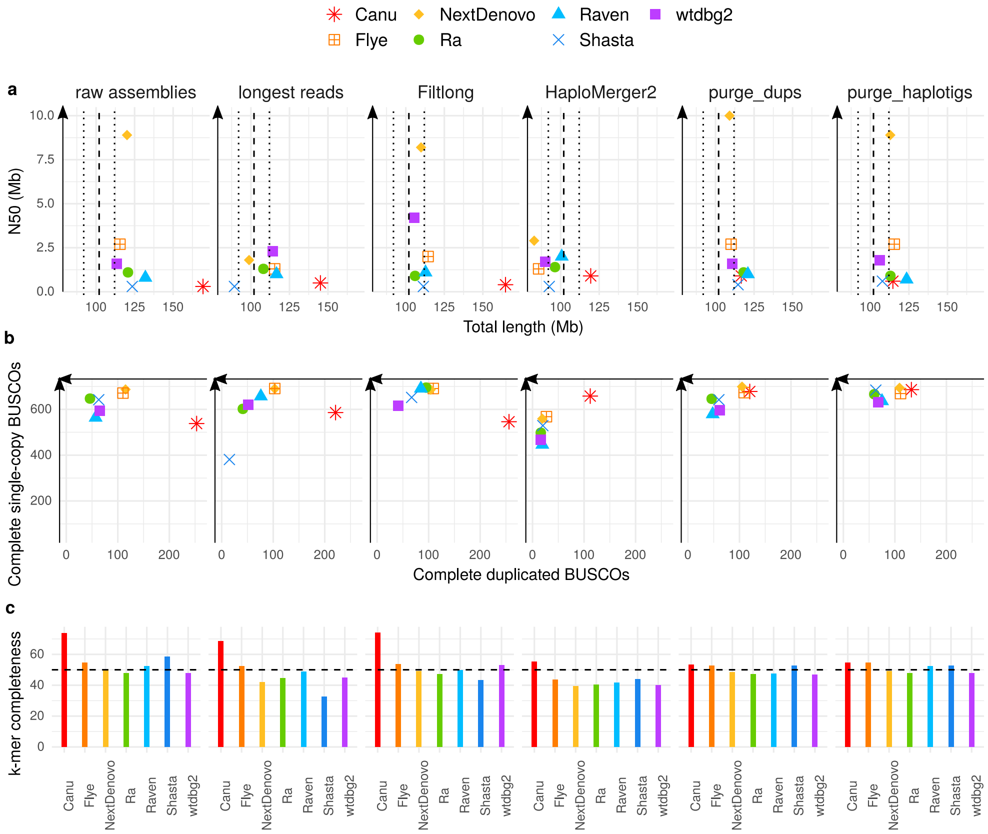

Figure S4. Statistics of raw assemblies obtained from the full PacBio dataset (raw assemblies), with a preliminary read filtering step (keeping only reads larger than 15 kb, or those selected by Filtlong based on quality and length) or a subsequent removal of uncollapsed haplotypes with HaploMerger2, purge_dups, or purge_haplotigs. a) N50 plotted against total assembly length. The dashed line indicates the expected genome size, with +/- 10 Mb margin delimited by the dotted lines. b) Number of complete single-copy BUSCOs plotted against number of complete duplicated BUSCOs, from a total of 954 orthologs. c) *k*-mer completeness. The dashed line indicates the expected 50% completeness.

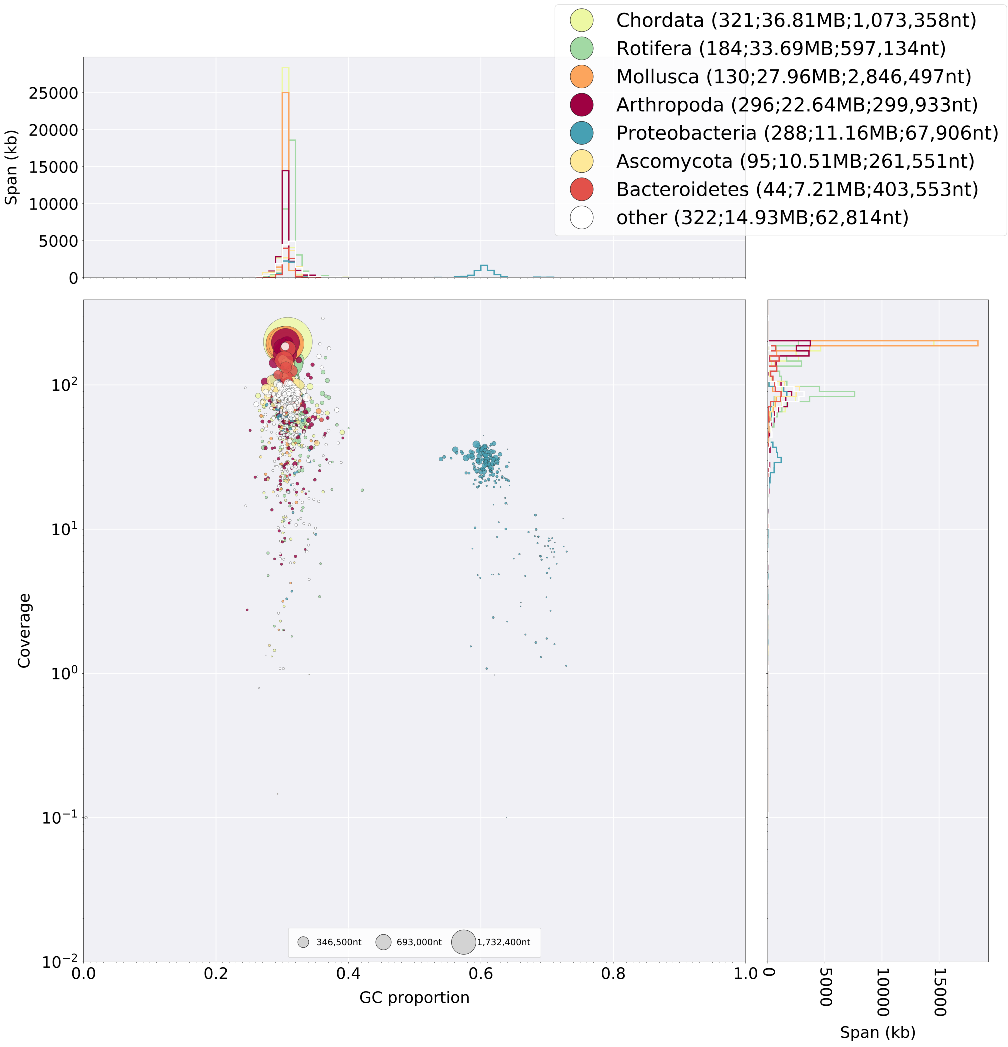

Figure S5. Blobtools v1.0 (Challis et al., 2020) analysis of a Canu assembly of the full PacBio dataset.

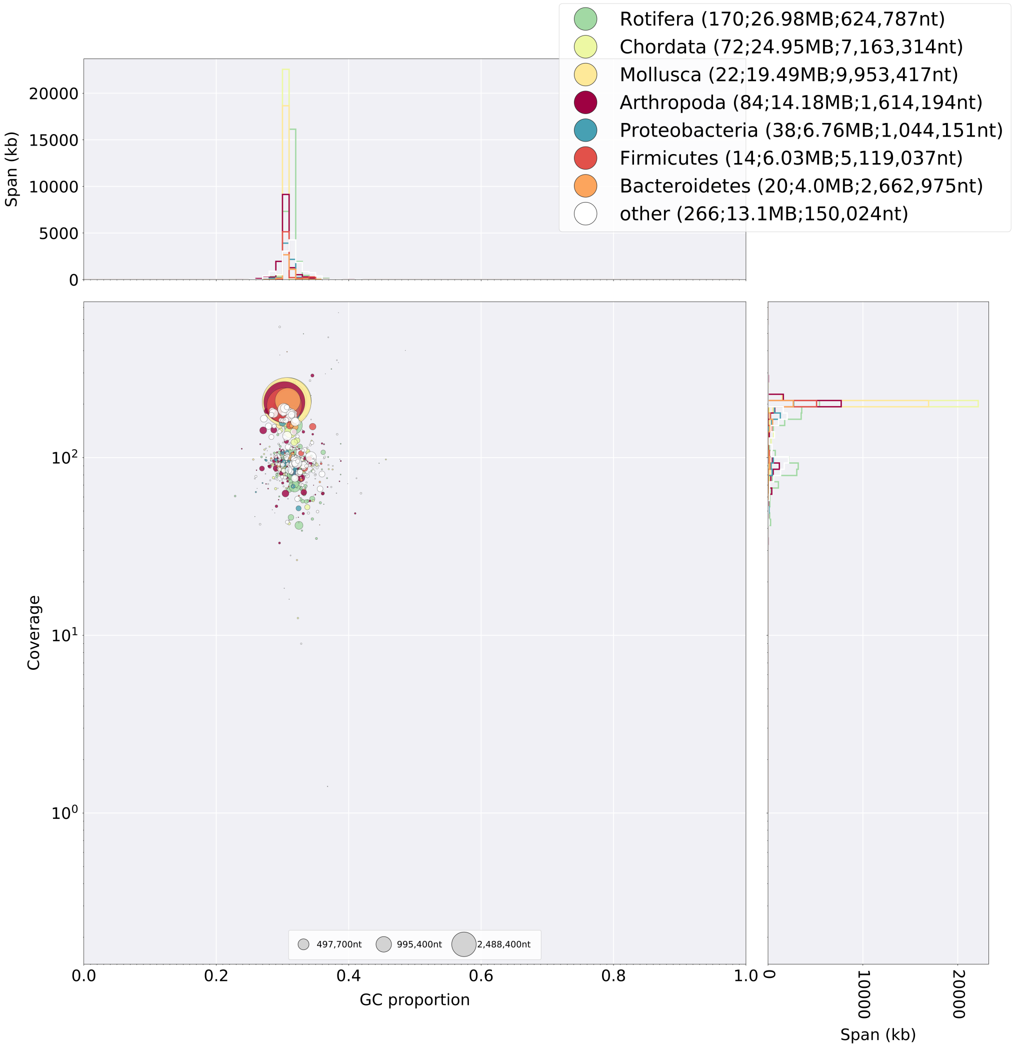

Figure S6. Blobtools v1.0 analysis of a Flye assembly of the full PacBio dataset.

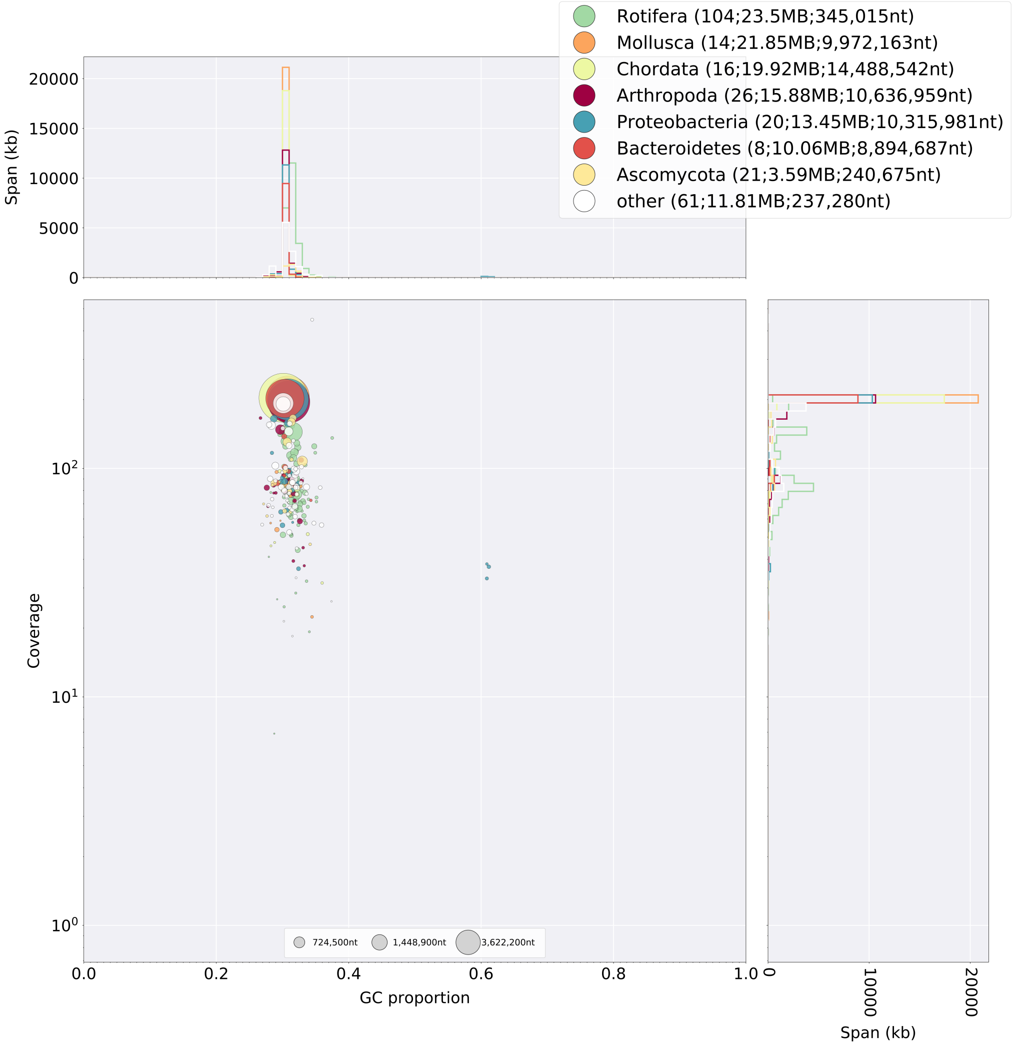

Figure S7. Blobtools v1.0 analysis of a NextDenovo assembly of the full PacBio dataset.

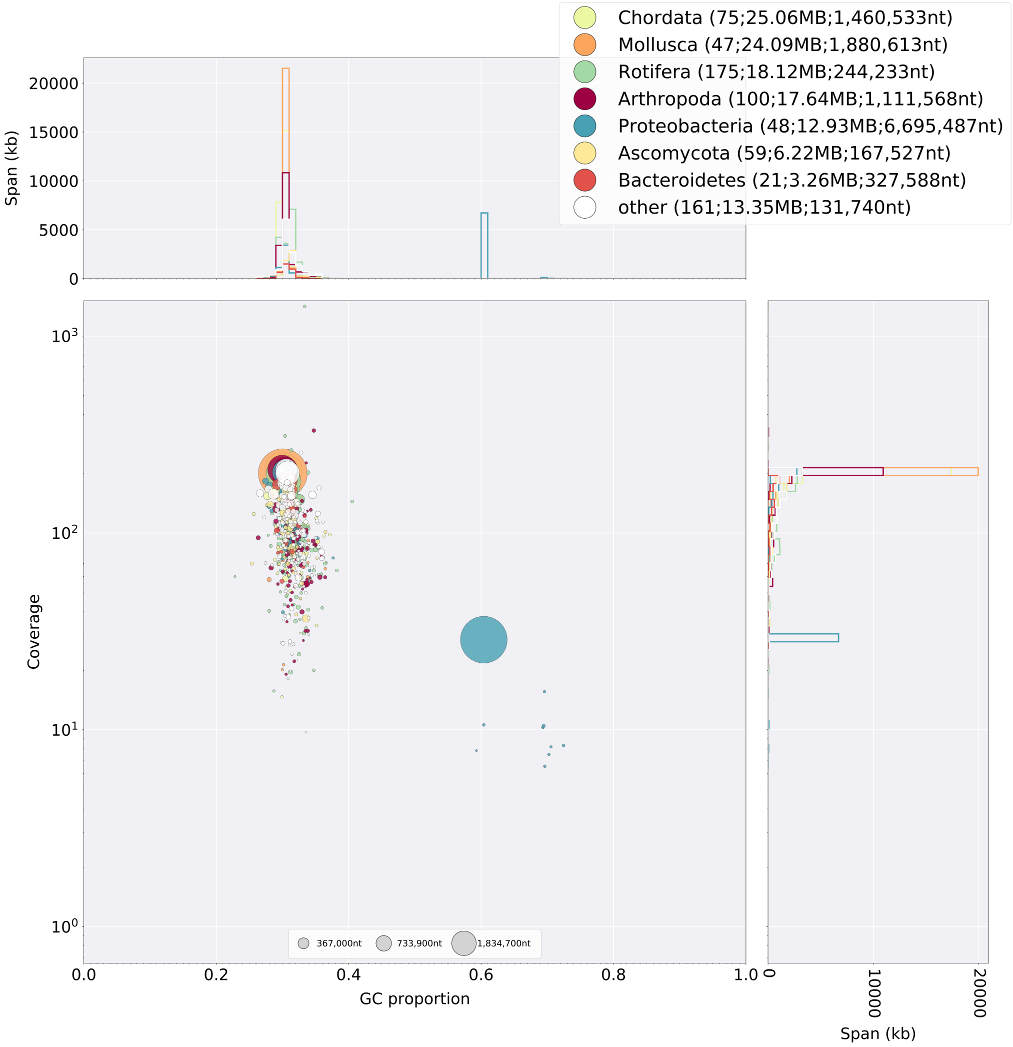

Figure S8. Blobtools v1.0 analysis of a Ra assembly of the full PacBio dataset.

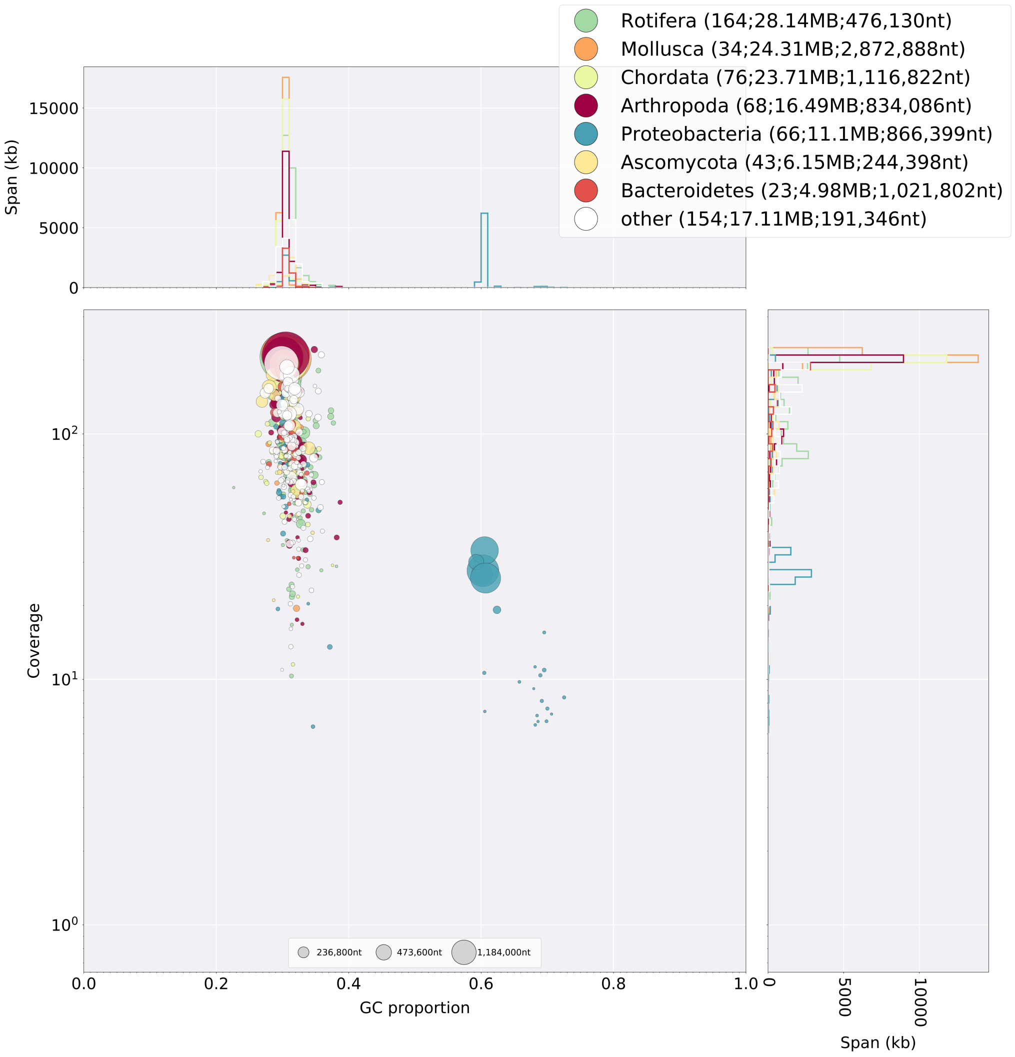

Figure S9. Blobtools v1.0 analysis of a Raven assembly of the full PacBio dataset.

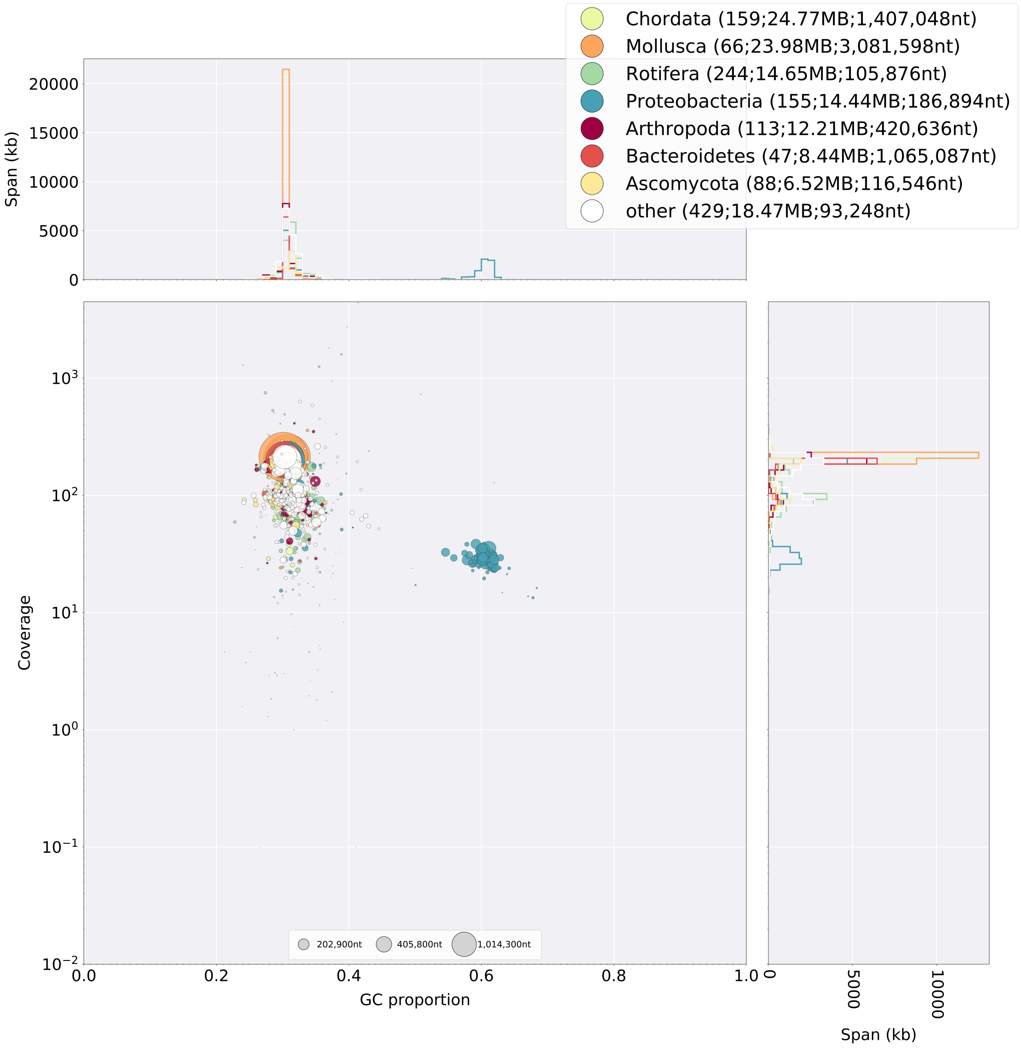

Figure S10. Blobtools v1.0 analysis of a Shasta assembly of the full PacBio dataset.

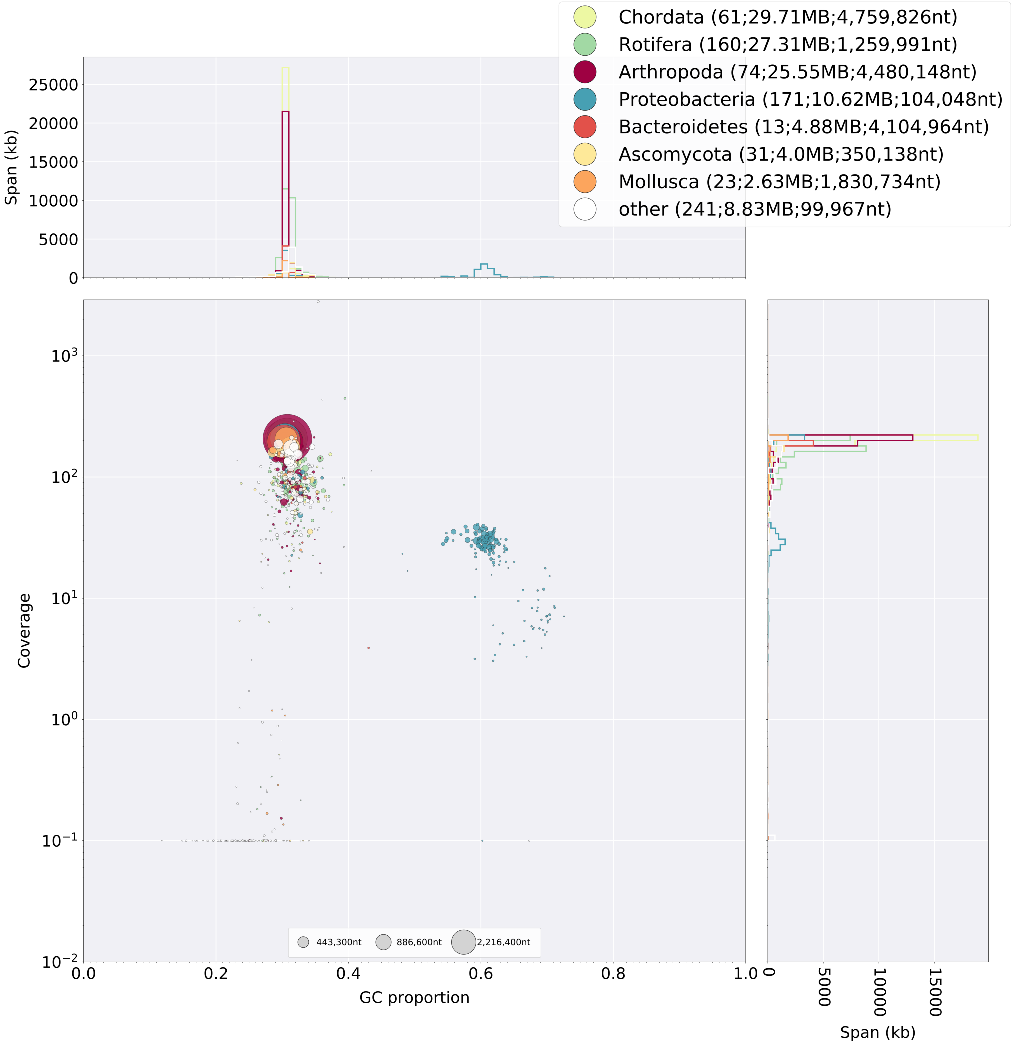

Figure S11. Blobtools v1.0 analysis of a wtdbg2 assembly of the full PacBio dataset.

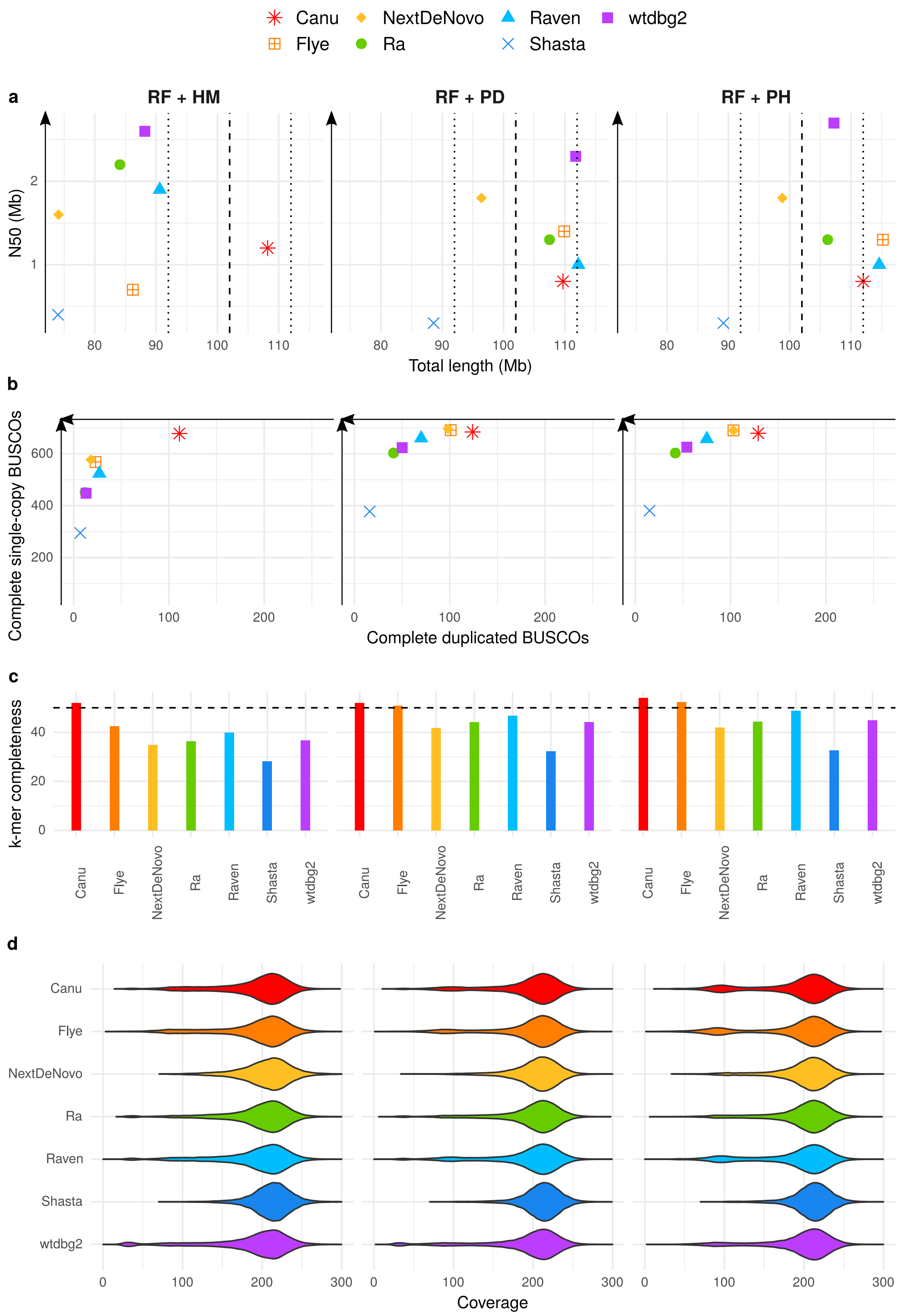

Figure S12. Statistics of PacBio assemblies obtained from the filtered PacBio dataset of reads longer than 15 kb, with a subsequent removal of uncollapsed haplotypes with HaploMerger2 (HM), purge_dups (PD), or purge_haplotigs (PH). a) N50 plotted against total assembly length. The dashed line indicates the expected genome size, with a +/- 10 Mb margin delimited by the dotted lines. b) Number of complete single-copy BUSCOs plotted against number of complete duplicated BUSCOs, from a total of 954 orthologs. c) *k*-mer completeness. The dashed line indicates the expected 50% completeness. d) Long-read coverage distribution over the contigs.

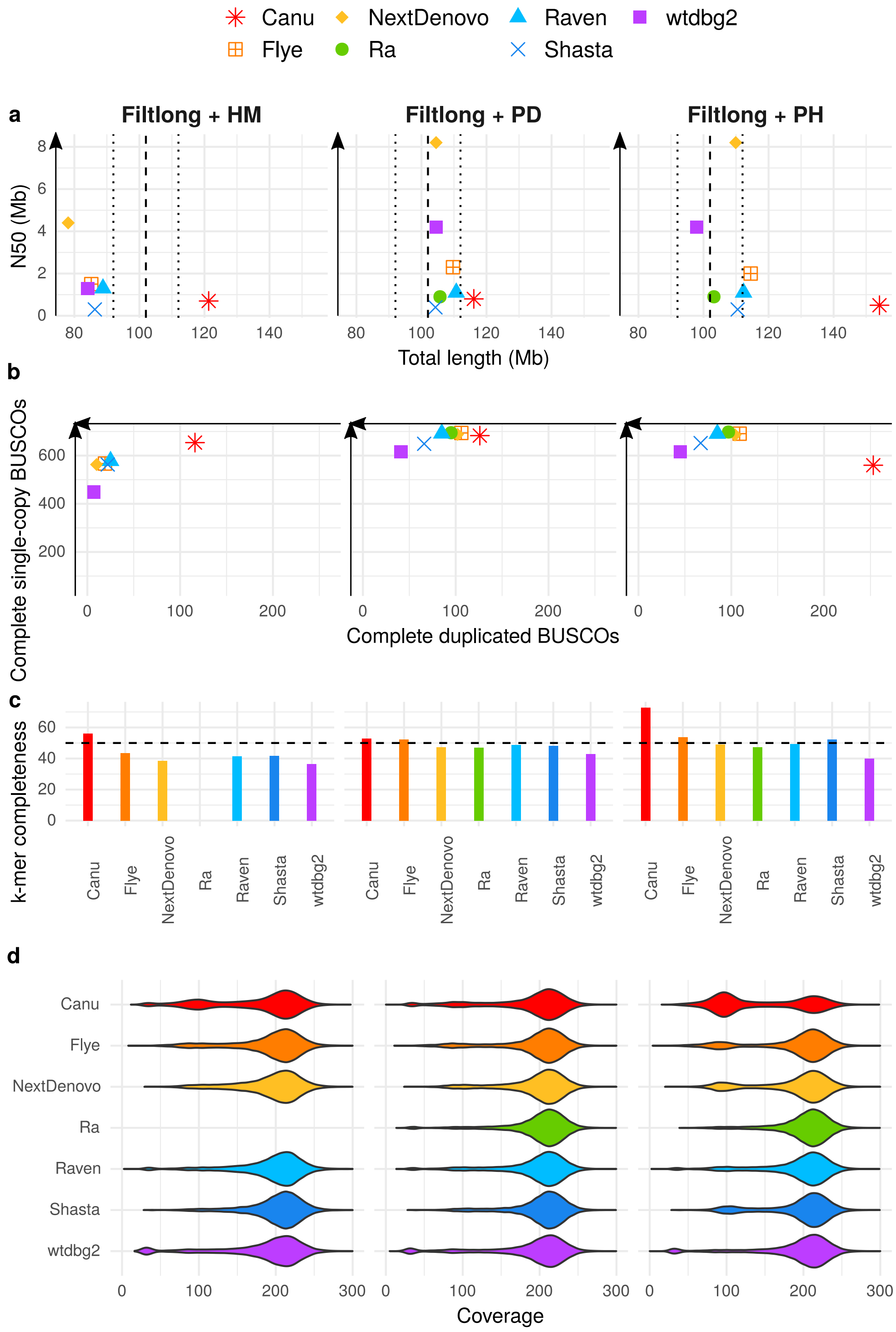

Figure S13. Statistics of PacBio assemblies obtained from the PacBio dataset filtered with Filtlong, with a subsequent removal of uncollapsed haplotypes with HaploMerger2 (HM), purge_dups (PD), or purge_haplotigs (PH). a) N50 plotted against total assembly length. The dashed line indicates the expected genome size, with a +/- 10 Mb margin delimited by the dotted lines. b) Number of complete single-copy BUSCOs plotted against number of complete duplicated BUSCOs, from a total of 954 orthologs. c) *k*-mer completeness. The dashed line indicates the expected 50% completeness. d) Long-read coverage distribution over the contigs.

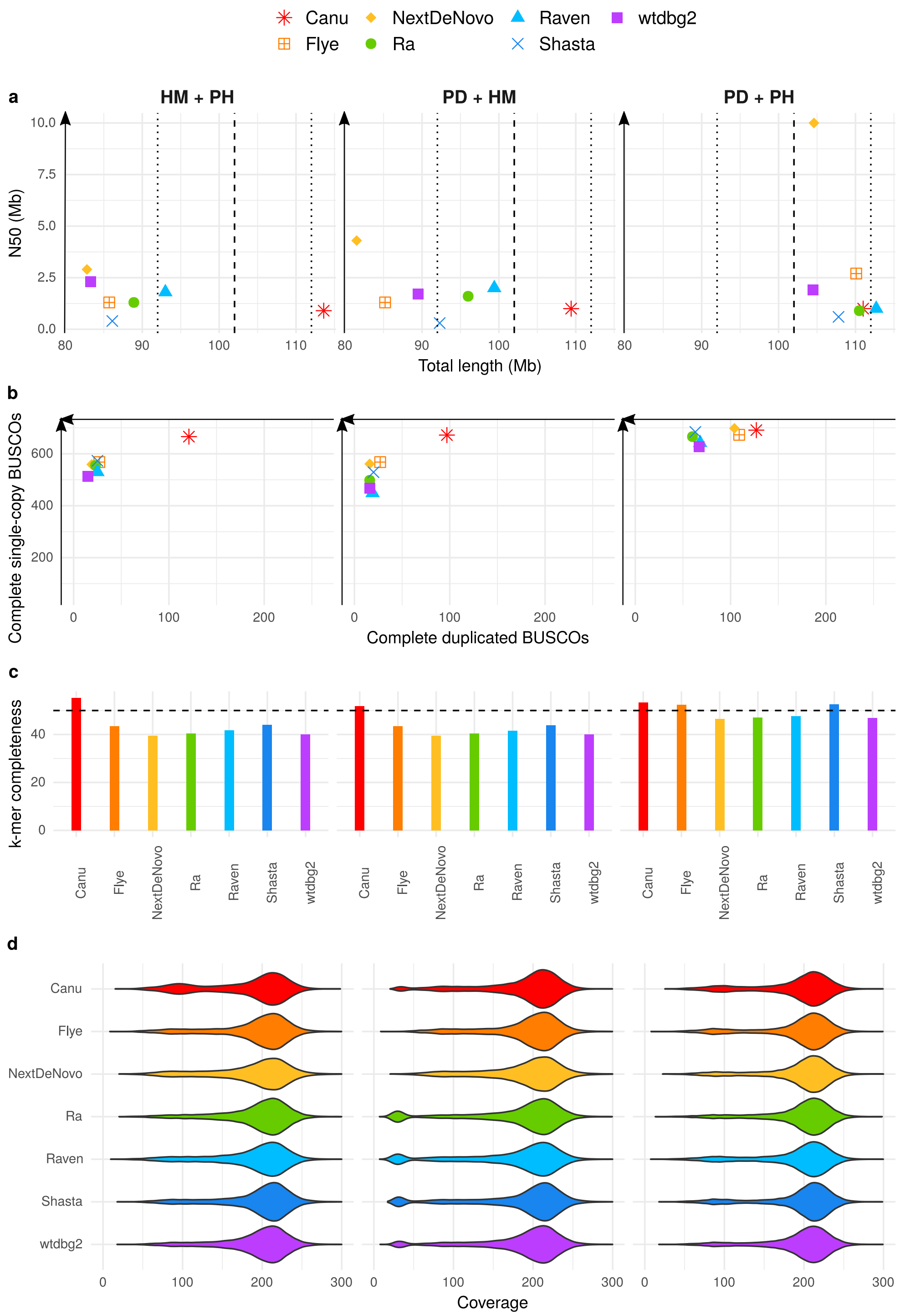

Figure S14. Statistics of PacBio assemblies obtained from the full PacBio dataset with a subsequent removal of uncollapsed haplotypes with combinations of HaploMerger2 (HM), purge_dups (PD), and purge_haplotigs (PH). a) N50 plotted against total assembly length. The dashed line indicates the expected genome size, with a +/- 10 Mb margin delimited by the dotted lines. b) Number of complete single-copy BUSCOs plotted against number of complete duplicated BUSCOs, from a total of 954 orthologs. c) *k*-mer completeness. The dashed line indicates the expected 50% completeness. d) Long-read coverage distribution over the contigs.

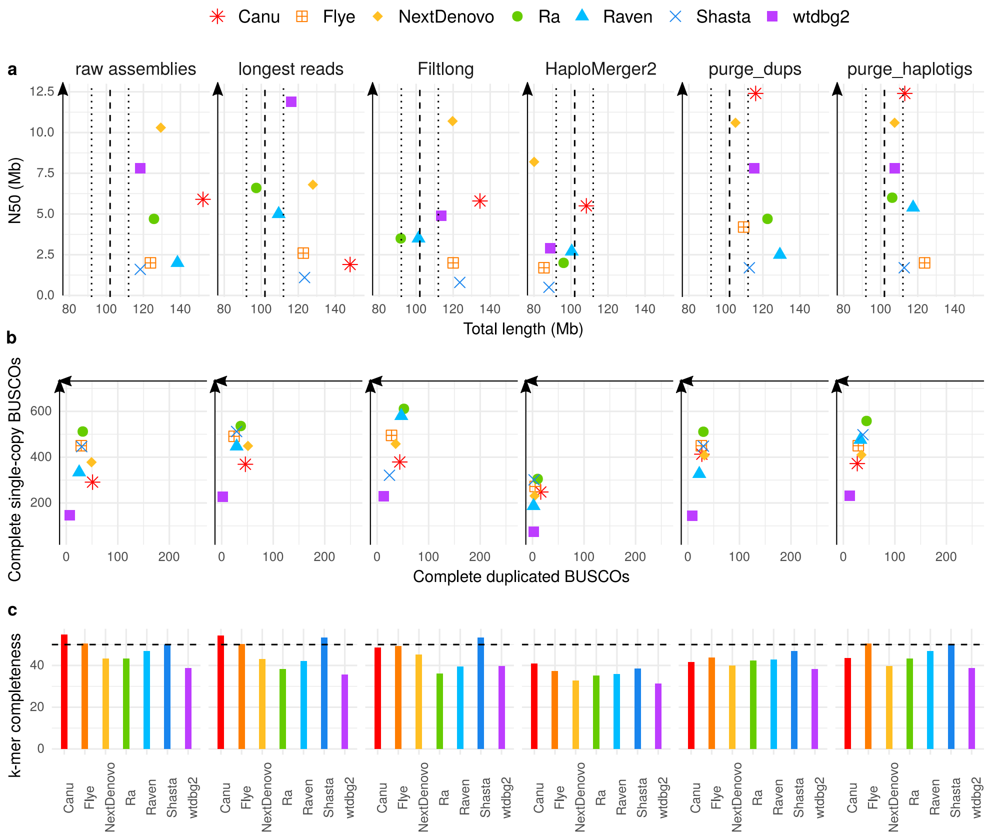

Figure S15. Statistics of raw assemblies obtained from the full Nanopore dataset (raw assemblies), with a preliminary read filtering step (keeping only reads larger than 30 kb, or those selected by Filtlong based on quality and length) or a subsequent removal of uncollapsed haplotypes with HaploMerger2, purge_dups, or purge_haplotigs. a) N50 plotted against total assembly length. The dashed line indicates the expected genome size, with +/- 10 Mb margin delimited by the dotted lines. b) Number of complete single-copy BUSCOs plotted against number of complete duplicated BUSCOs, from a total of 954 orthologs. c) *k*-mer completeness. The dashed line indicates the expected 50% completeness.

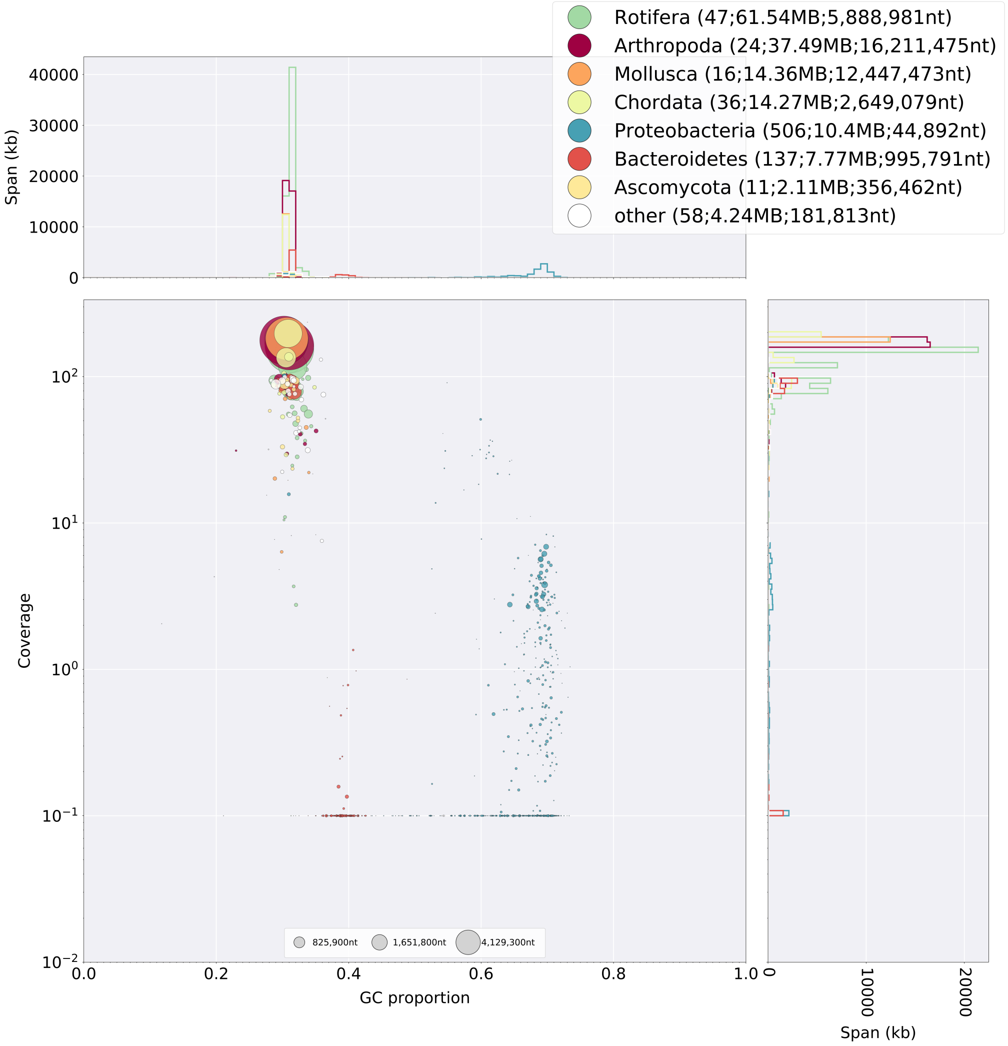

Figure S16. Blobtools v1.0 analysis of a Canu assembly of the full Nanopore dataset.

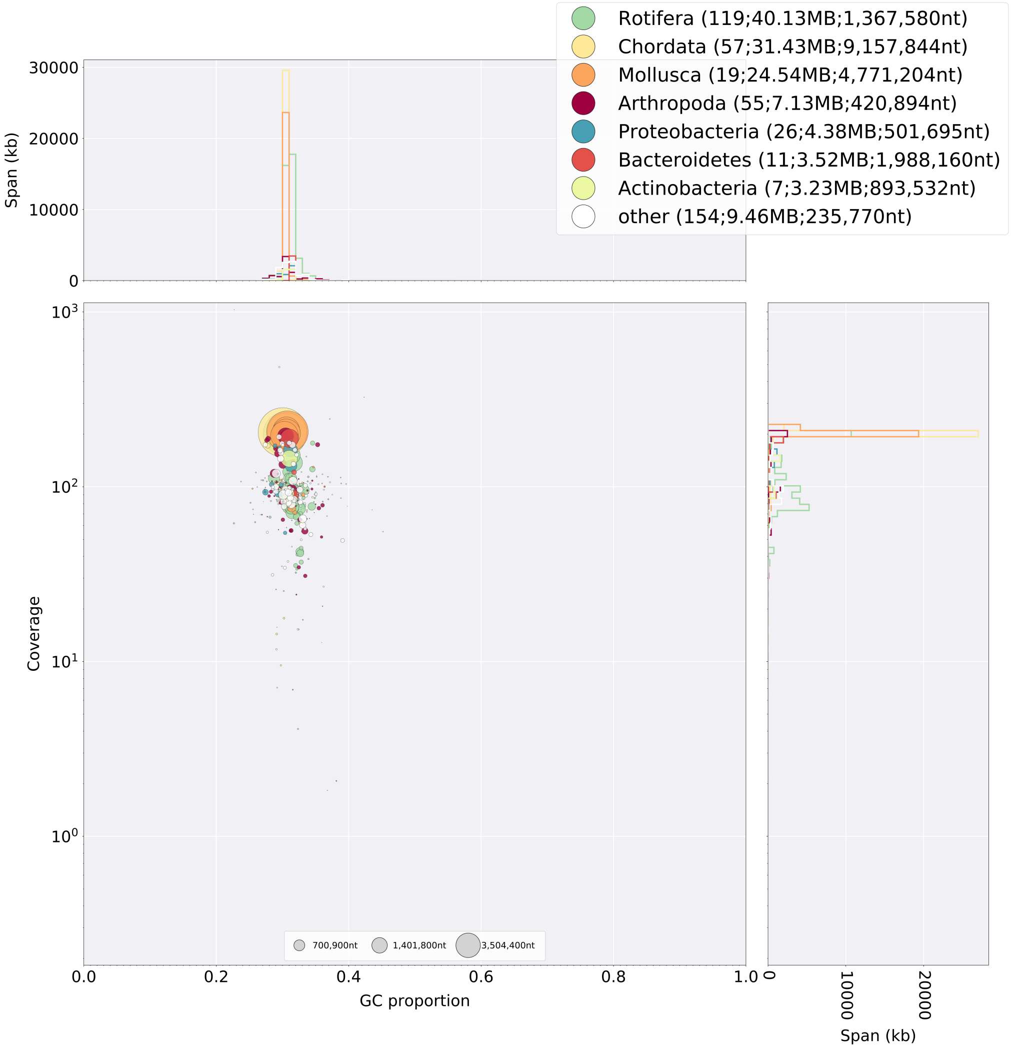

Figure S17. Blobtools v1.0 analysis of a Flye assembly of the full Nanopore dataset.

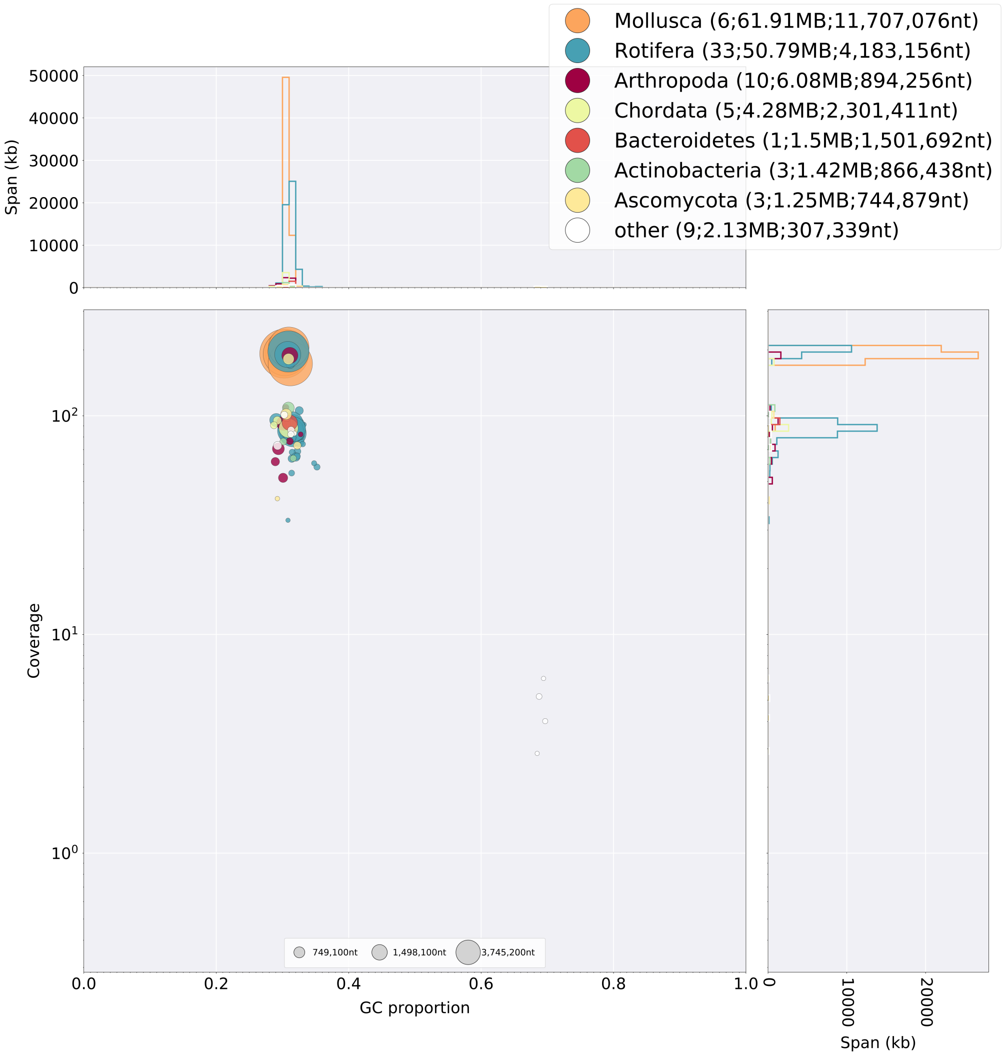

Figure S18. Blobtools v1.0 analysis of a NextDenovo assembly of the full Nanopore dataset.

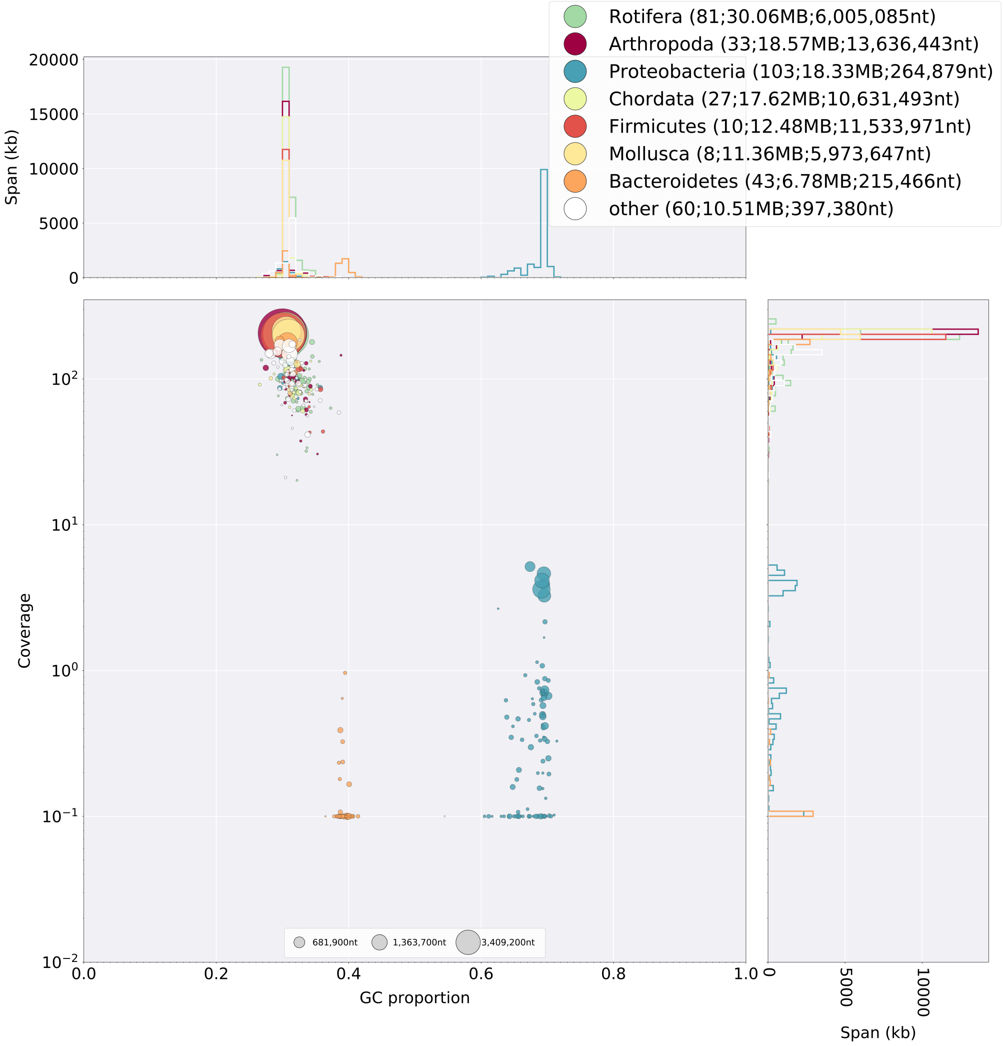

Figure S19. Blobtools v1.0 analysis of a Ra assembly of the full Nanopore dataset.

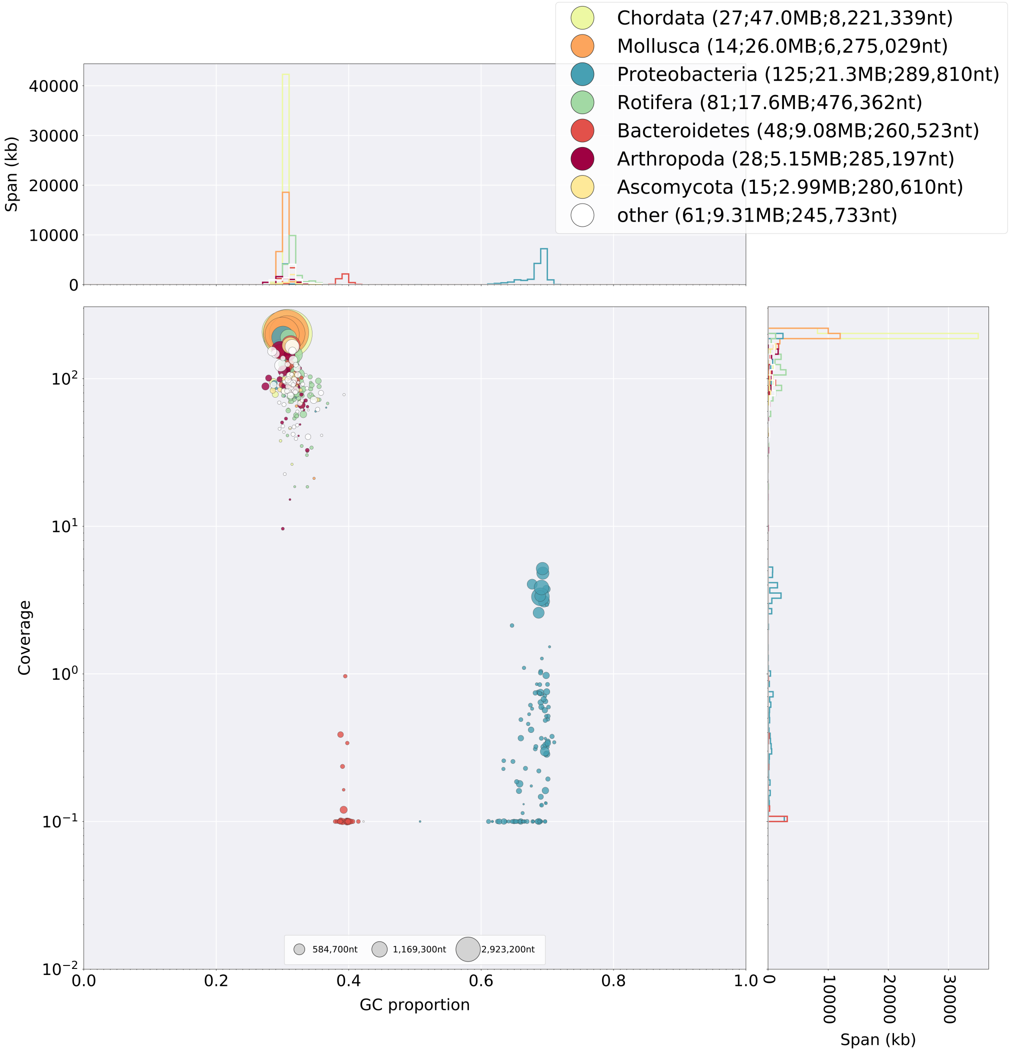

Figure S20. Blobtools v1.0 analysis of a Raven assembly of the full Nanopore dataset.

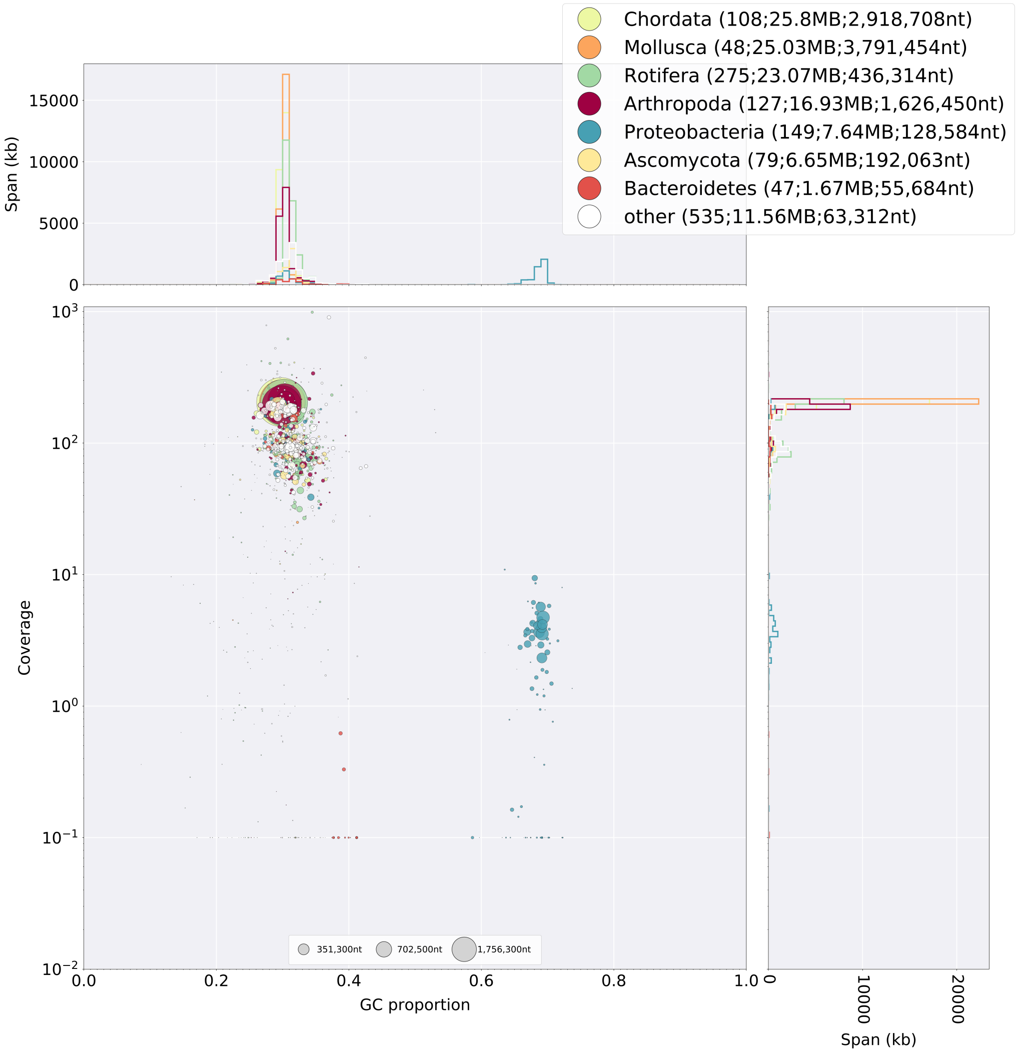

Figure S21. Blobtools v1.0 analysis of a Shasta assembly of the full Nanopore dataset.

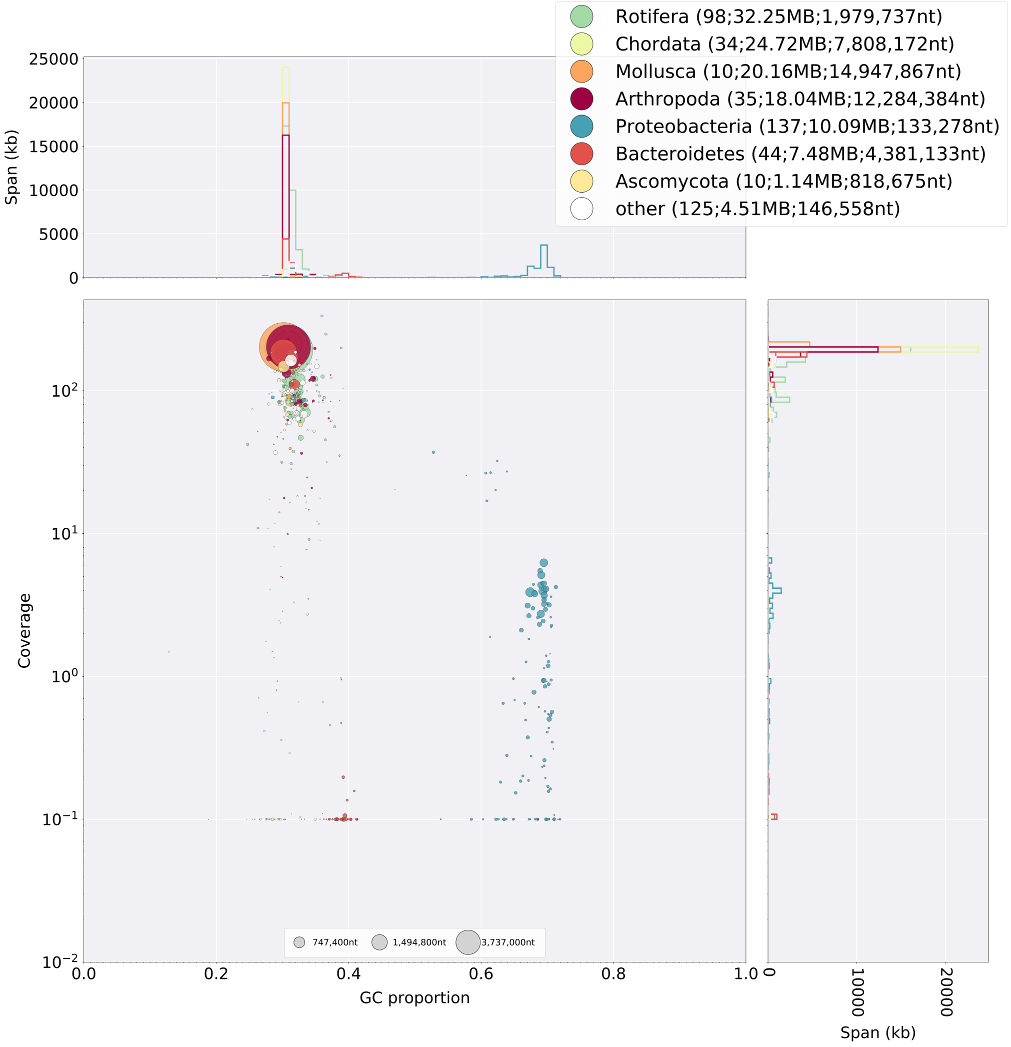

Figure S22. Blobtools v1.0 analysis of a wtdbg2 assembly of the full Nanopore dataset.

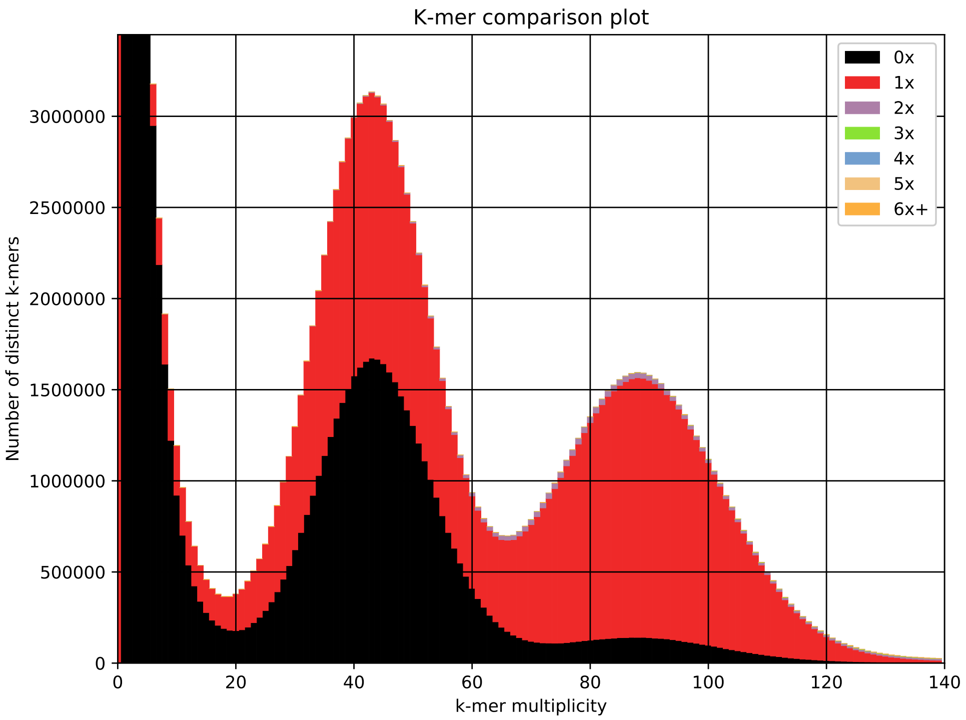

Figure S23. *k*-mer spectrum of the Shasta assembly of the full Nanopore dataset obtained with KAT v2.4.2.

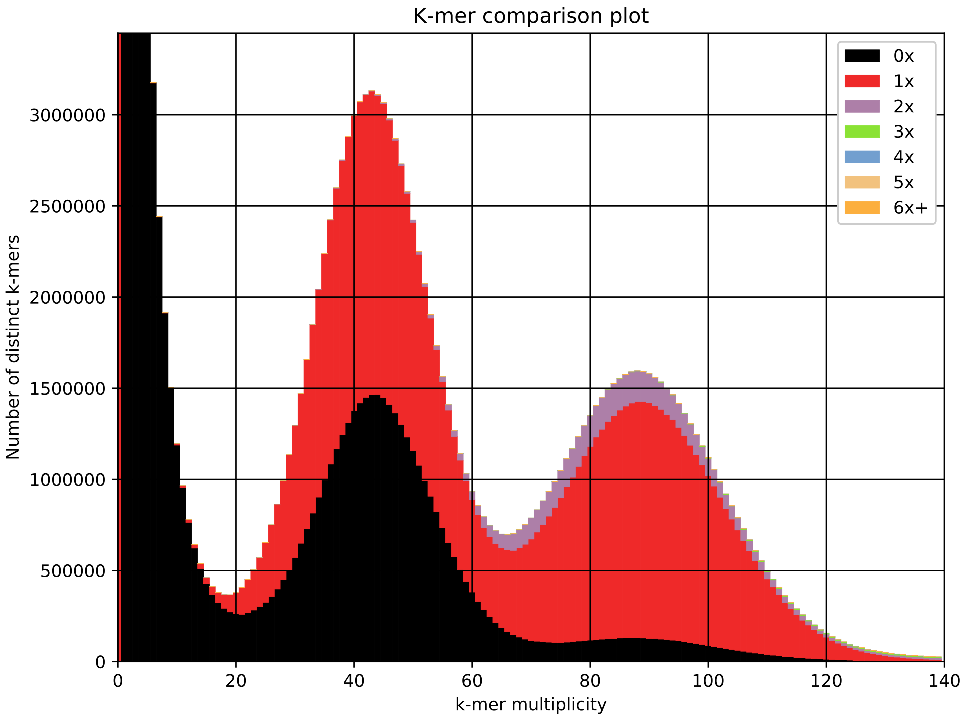

Figure S24. *k*-mer spectrum of the Shasta assembly of the longest Nanopore reads obtained with KAT v2.4.2.

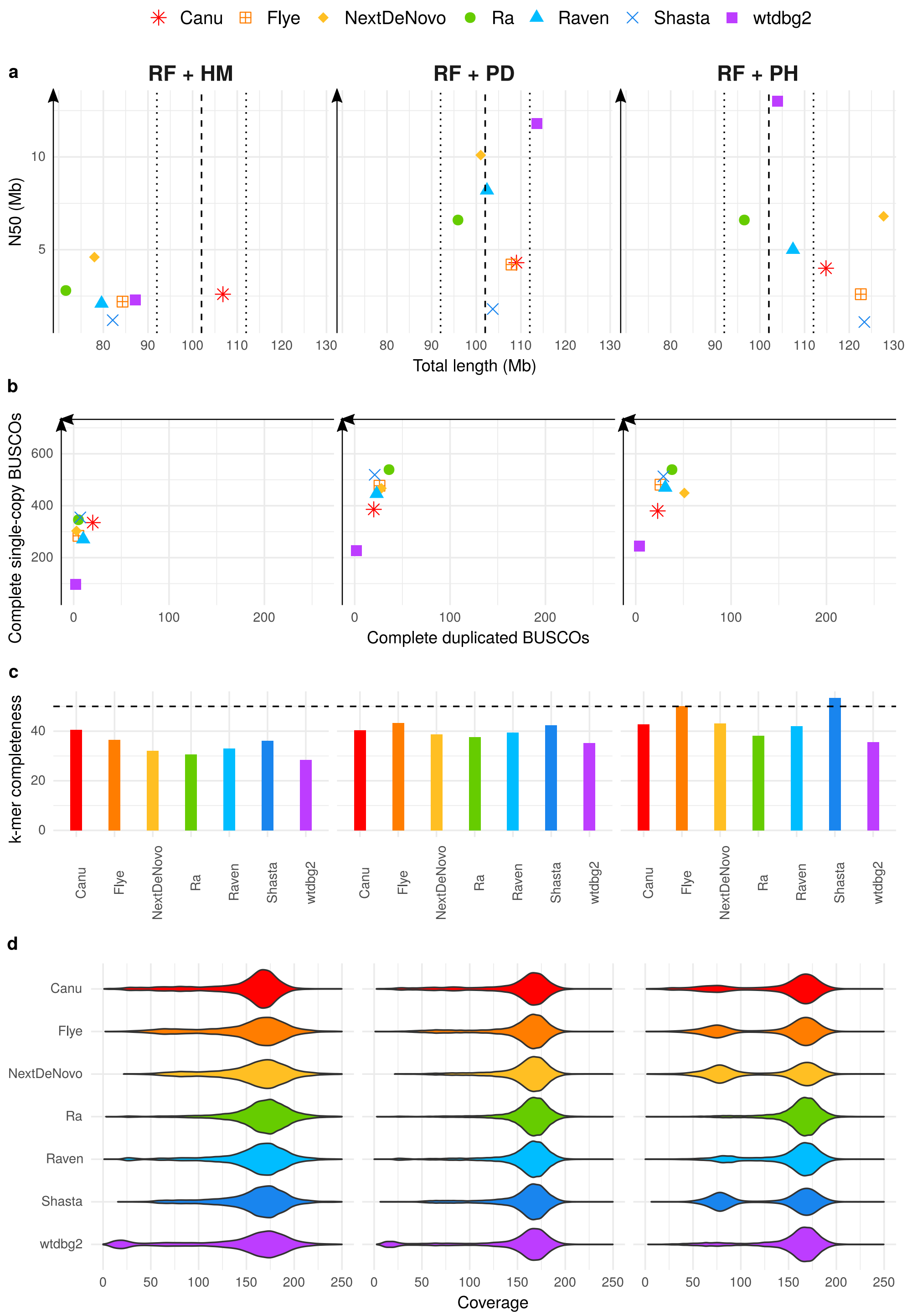

Figure S25. Statistics of Nanopore assemblies obtained from the filtered Nanopore dataset of reads longer than 30 kb, with a subsequent removal of uncollapsed haplotypes with HaploMerger2 (HM), purge_dups (PD), or purge_haplotigs (PH). a) N50 plotted against total assembly length. The dashed line indicates the expected genome size, with a +/- 10 Mb margin delimited by the dotted lines. b) Number of complete single-copy BUSCOs plotted against number of complete duplicated BUSCOs, from a total of 954 orthologs. c) *k*-mer completeness. The dashed line indicates the expected 50% completeness. d) Long-read coverage distribution over the contigs.

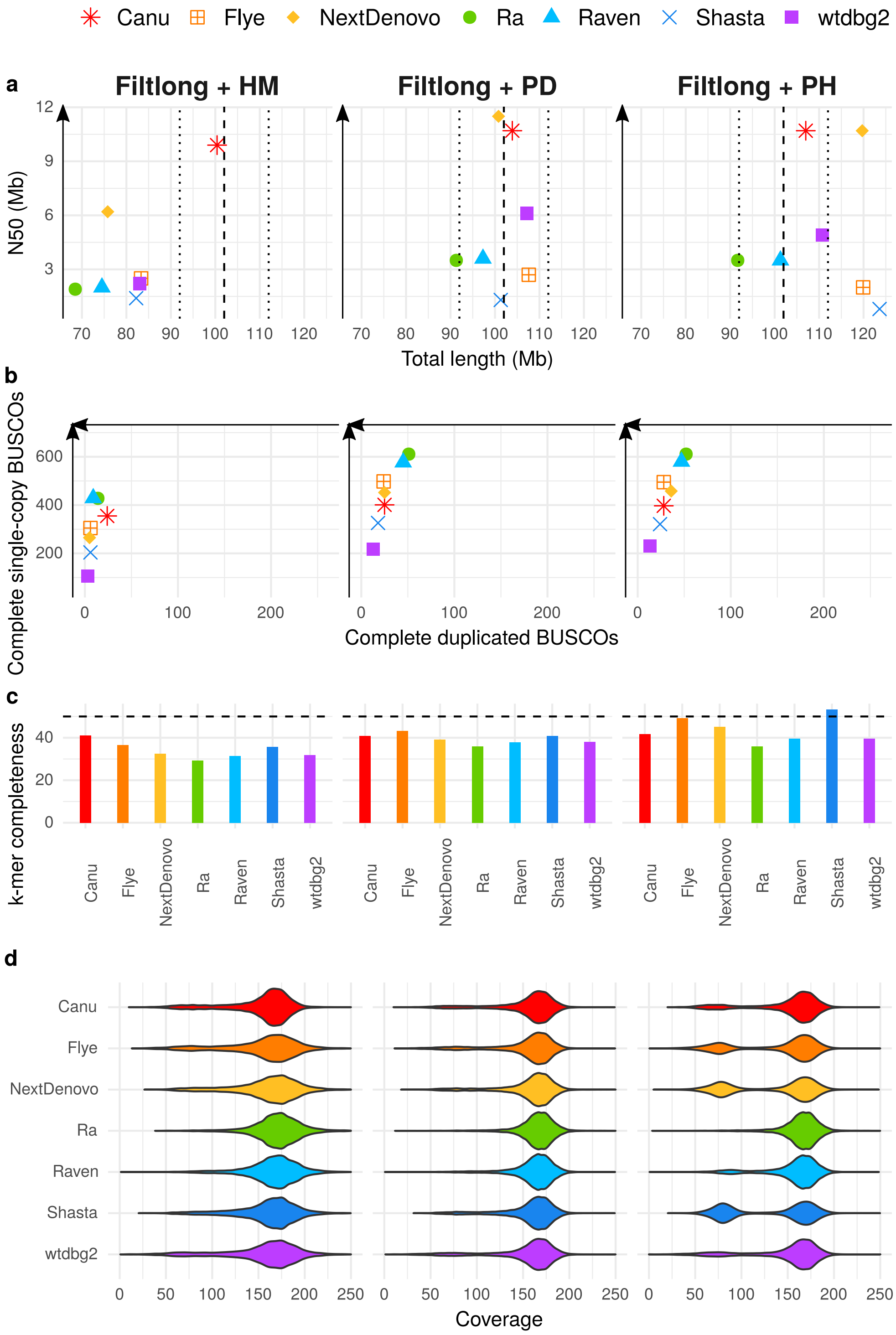

Figure S26. Statistics of Nanopore assemblies obtained from the Nanopore dataset filtered with Filtlong, with a subsequent removal of uncollapsed haplotypes with HaploMerger2 (HM), purge_dups (PD), or purge_haplotigs (PH). a) N50 plotted against total assembly length. The dashed line indicates the expected genome size, with a +/- 10 Mb margin delimited by the dotted lines. b) Number of complete single-copy BUSCOs plotted against number of complete duplicated BUSCOs, from a total of 954 orthologs. c) *k*-mer completeness. The dashed line indicates the expected 50% completeness. d) Long-read coverage distribution over the contigs.

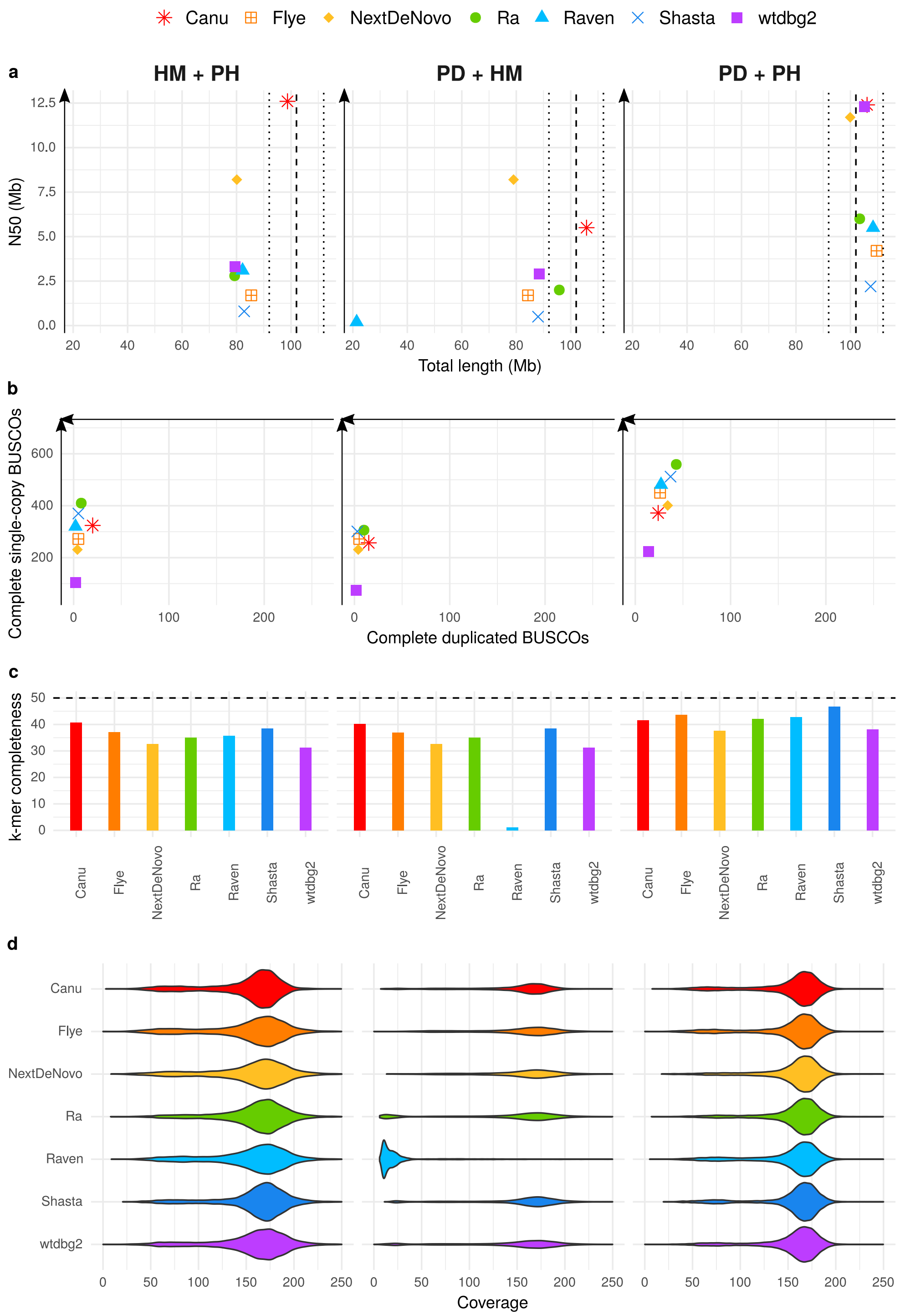

Figure S27. Statistics of Nanopore assemblies obtained from the full Nanopore dataset with a subsequent removal of uncollapsed haplotypes with combinations of HaploMerger2 (HM), purge_dups (PD), and purge_haplotigs (PH). a) N50 plotted against total assembly length. The dashed line indicates the expected genome size, with a +/- 10 Mb margin delimited by the dotted lines. b) Number of complete single-copy BUSCOs plotted against number of complete duplicated BUSCOs, from a total of 954 orthologs. c) *k*-mer completeness. The dashed line indicates the expected 50% completeness. d) Long-read coverage distribution over the contigs.

Table S1. Haploidy values computed by HapPy v0.1 for PacBio assemblies.

Assembler	Processing	Haploidy
Canu	raw assemblies	0.59
Flye	raw assemblies	0.85
NextDenovo	raw assemblies	0.81
Ra	raw assemblies	0.90
Raven	raw assemblies	0.82
Shasta	raw assemblies	0.83
wtdbg2	raw assemblies	0.90
Canu	longest reads	0.62
Flye	longest reads	0.85
NextDenovo	longest reads	0.94
Ra	longest reads	0.94
Raven	longest reads	0.88
Shasta	longest reads	0.96
wtdbg2	longest reads	0.90
Canu	Filtlong	0.58
Flye	Filtlong	0.86
NextDenovo	Filtlong	0.88
Ra	Filtlong	0.94
Raven	Filtlong	0.90
Shasta	Filtlong	0.85
wtdbg2	Filtlong	0.91
Canu	HaploMerger2	0.84
Flye	HaploMerger2	0.89
NextDenovo	HaploMerger2	0.88
Ra	HaploMerger2	0.92
Raven	HaploMerger2	0.90
Shasta	HaploMerger2	0.91
wtdbg2	HaploMerger2	0.92
Canu	purge_dups	0.89
Flye	purge_dups	0.89
NextDenovo	purge_dups	0.90
Ra	purge_dups	0.91
Raven	purge_dups	0.90
Shasta	purge_dups	0.90
wtdbg2	purge_dups	0.91
Canu	purge_haplotigs	0.86
Flye	purge_haplotigs	0.85
NextDenovo	purge_haplotigs	0.87
Ra	purge_haplotigs	0.88
Raven	purge_haplotigs	0.80
Shasta	purge_haplotigs	0.90
wtdbg2	purge_haplotigs	0.90

Table S2. Haploidy values computed by HapPy v0.1 for PacBio assemblies.

Assembler	Processing	Haploidy
Canu	longest reads + purge_haplotigs	0.87
Flye	longest reads + purge_haplotigs	0.85
NextDenovo	longest reads + purge_haplotigs	0.94
Ra	longest reads + purge_haplotigs	0.92
Raven	longest reads + purge_haplotigs	0.87
Shasta	longest reads + purge_haplotigs	0.96
wtdbg2	longest reads + purge_haplotigs	0.90
Canu	longest reads + purge_dups	0.91
Flye	longest reads + purge_dups	0.90
NextDenovo	longest reads + purge_dups	0.97
Ra	longest reads + purge_dups	0.95
Raven	longest reads + purge_dups	0.91
Shasta	longest reads + purge_dups	0.97
wtdbg2	longest reads + purge_dups	0.92
Canu	Filtlong + purge_haplotigs	0.56
Flye	Filtlong + purge_haplotigs	0.86
NextDenovo	Filtlong + purge_haplotigs	0.88
Ra	Filtlong + purge_haplotigs	0.93
Raven	Filtlong + purge_haplotigs	0.90
Shasta	Filtlong + purge_haplotigs	0.85
wtdbg2	Filtlong + purge_haplotigs	0.94
Canu	Filtlong + purge_dups	0.90
Flye	Filtlong + purge_dups	0.90
NextDenovo	Filtlong + purge_dups	0.93
Ra	Filtlong + purge_dups	0.94
Raven	Filtlong + purge_dups	0.92
Shasta	Filtlong + purge_dups	0.92
wtdbg2	Filtlong + purge_dups	0.91

Table S3. Haploidy values computed by HapPy v0.1 for PacBio assemblies.

Assembler	Processing	Haploidy
Canu	HaploMerger2 + purge_haplotigs	0.82
Flye	HaploMerger2 + purge_haplotigs	0.89
NextDenovo	HaploMerger2 + purge_haplotigs	0.88
Ra	HaploMerger2 + purge_haplotigs	0.88
Raven	HaploMerger2 + purge_haplotigs	0.83
Shasta	HaploMerger2 + purge_haplotigs	0.88
wtdbg2	HaploMerger2 + purge_haplotigs	0.84
Canu	purge_dups + HaploMerger2	0.91
Flye	purge_dups + HaploMerger2	0.90
NextDenovo	purge_dups + HaploMerger2	0.90
Ra	purge_dups + HaploMerger2	0.92
Raven	purge_dups + HaploMerger2	0.93
Shasta	purge_dups + HaploMerger2	0.92
wtdbg2	purge_dups + HaploMerger2	0.92
Canu	purge_dups + purge_haplotigs	0.88
Flye	purge_dups + purge_haplotigs	0.89
NextDenovo	purge_dups + purge_haplotigs	0.92
Ra	purge_dups + purge_haplotigs	0.89
Raven	purge_dups + purge_haplotigs	0.88
Shasta	purge_dups + purge_haplotigs	0.90
wtdbg2	purge_dups + purge_haplotigs	0.91

Table S4. Haploidy values computed by HapPy v0.1 for Nanopore assemblies.

Assembler	Processing	Haploidy
Canu	raw assemblies	0.63
Flye	raw assemblies	0.79
NextDenovo	raw assemblies	0.72
Ra	raw assemblies	0.90
Raven	raw assemblies	0.83
Shasta	raw assemblies	0.86
wtdbg2	raw assemblies	0.92
Canu	longest reads	0.59
Flye	longest reads	0.79
NextDenovo	longest reads	0.72
Ra	longest reads	0.95
Raven	longest reads	0.89
Shasta	longest reads	0.75
wtdbg2	longest reads	0.92
Canu	Filtlong	0.67
Flye	Filtlong	0.81
NextDenovo	Filtlong	0.77
Ra	Filtlong	0.97
Raven	Filtlong	0.92
Shasta	Filtlong	0.72
wtdbg2	Filtlong	0.87
Canu	HaploMerger2	0.89
Flye	HaploMerger2	0.87
NextDenovo	HaploMerger2	0.89
Ra	HaploMerger2	0.91
Raven	HaploMerger2	0.88
Shasta	HaploMerger2	0.90
wtdbg2	HaploMerger2	0.89
Canu	purge_dups	0.92
Flye	purge_dups	0.90
NextDenovo	purge_dups	0.92
Ra	purge_dups	0.93
Raven	purge_dups	0.90
Shasta	purge_dups	0.91
wtdbg2	purge_dups	0.93
Canu	purge_haplotigs	0.86
Flye	purge_haplotigs	0.79
NextDenovo	purge_haplotigs	0.90
Ra	purge_haplotigs	0.90
Raven	purge_haplotigs	0.83
Shasta	purge_haplotigs	0.86
wtdbg2	purge_haplotigs	0.91

Table S5. Haploidy values computed by HapPy v0.1 for Nanopore assemblies.

Assembler	Processing	Haploidy
Canu	longest reads + purge_haplotigs	0.85
Flye	longest reads + purge_haplotigs	0.79
NextDenovo	longest reads + purge_haplotigs	0.72
Ra	longest reads + purge_haplotigs	0.95
Raven	longest reads + purge_haplotigs	0.89
Shasta	longest reads + purge_haplotigs	0.75
wtdbg2	longest reads + purge_haplotigs	0.91
Canu	longest reads + purge_dups	0.89
Flye	longest reads + purge_dups	0.91
NextDenovo	longest reads + purge_dups	0.95
Ra	longest reads + purge_dups	0.96
Raven	longest reads + purge_dups	0.95
Shasta	longest reads + purge_dups	0.93
wtdbg2	longest reads + purge_dups	0.92
Canu	Filtlong + purge_haplotigs	0.90
Flye	Filtlong + purge_haplotigs	0.81
NextDenovo	Filtlong + purge_haplotigs	0.77
Ra	Filtlong + purge_haplotigs	0.97
Raven	Filtlong + purge_haplotigs	0.92
Shasta	Filtlong + purge_haplotigs	0.72
wtdbg2	Filtlong + purge_haplotigs	0.89
Canu	Filtlong + purge_dups	0.93
Flye	Filtlong + purge_dups	0.91
NextDenovo	Filtlong + purge_dups	0.94
Ra	Filtlong + purge_dups	0.97
Raven	Filtlong + purge_dups	0.96
Shasta	Filtlong + purge_dups	0.94
wtdbg2	Filtlong + purge_dups	0.91

Table S6. Haploidy values computed by HapPy v0.1 for Nanopore assemblies.

Assembler	Processing	Haploidy
Canu	HaploMerger2 + purge_haplotigs	0.89
Flye	HaploMerger2 + purge_haplotigs	0.87
NextDenovo	HaploMerger2 + purge_haplotigs	0.89
Ra	HaploMerger2 + purge_haplotigs	0.91
Raven	HaploMerger2 + purge_haplotigs	0.92
Shasta	HaploMerger2 + purge_haplotigs	0.90
wtdbg2	HaploMerger2 + purge_haplotigs	0.90
Canu	purge_dups + purge_haplotigs	0.91
Flye	purge_dups + purge_haplotigs	0.90
NextDenovo	purge_dups + purge_haplotigs	0.94
Ra	purge_dups + purge_haplotigs	0.93
Raven	purge_dups + purge_haplotigs	0.90
Shasta	purge_dups + purge_haplotigs	0.91
wtdbg2	purge_dups + purge_haplotigs	0.92
Canu	purge_dups + HaploMerger2	0.90
Flye	purge_dups + HaploMerger2	0.88
NextDenovo	purge_dups + HaploMerger2	0.90
Ra	purge_dups + HaploMerger2	0.91
Raven	purge_dups + HaploMerger2	0.51
Shasta	purge_dups + HaploMerger2	0.90
wtdbg2	purge_dups + HaploMerger2	0.89

Table S7. List of command lines used for each tool. Values L, M, H for `purge_haplotigs cov` were selected for each assembly according to the histogram produced by `purge_haplotigs hist`.

<table>
<tr><th>Program</th><th>Dataset</th><th>Command lines</th></tr>
<tr><td>Filtlong</td><td>-</td><td>filtlong --target_bases 4092000000 --mean_q_weight 10 long_read_data</td></tr>
<tr><td>Canu</td><td>PacBio</td><td>canu -d out -p out genomeSize=100m useGrid=false -pacbio-raw pb_data</td></tr>
<tr><td>Canu</td><td>Nanopore</td><td>canu -d out -p out genomeSize=100m useGrid=false -nanopore-raw ont_data</td></tr>
<tr><td>Flye</td><td>PacBio</td><td>flye -o out -g 100m --pacbio-raw pb_data</td></tr>
<tr><td>Flye</td><td>Nanopore</td><td>flye -o out -g 100m --nano-raw ont_data</td></tr>
<tr><td>NextDenovo</td><td>PacBio</td><td>echo pb_data > input.fofn
seq_stat input.fofn -g 100Mb -d 150 > stats.txt
NextDenovo run.cfg</td></tr>
<tr><td>NextDenovo</td><td>Nanopore</td><td>echo ont_data > input.fofn
seq_stat input.fofn -g 100Mb -d 150 > stats.txt
NextDenovo run.cfg</td></tr>
<tr><td>Ra</td><td>PacBio</td><td>ra -x pb pb_data > assembly.fasta</td></tr>
<tr><td>Ra</td><td>Nanopore</td><td>ra -x ont ont_data > assembly.fasta</td></tr>
<tr><td>Raven</td><td>-</td><td>raven long_read_data > assembly.fasta</td></tr>
<tr><td>Shasta</td><td>PacBio</td><td>shasta --input pb_data --Reads.minReadLength 0 --assemblyDirectory out --Assembly.consensusCaller Modal --Kmers.k 12</td></tr>
<tr><td>Shasta</td><td>Nanopore</td><td>shasta --input ont_data --Reads.minReadLength 0 --assemblyDirectory out</td></tr>
<tr><td>wtdbg2</td><td>PacBio</td><td>wtdbg2 -x rs -g 100m -i pb_data -fo out
wtpoa-cns -i out.ctg.lay.gz -o out.ctg.fa
minimap2 -x map-pb -a out.ctg.fa pb_data | samtools sort > out.ctg.bam
samtools view out.ctg.bam | wtpoa-cns -d out.ctg.fa -i - -fo assembly.fasta</td></tr>
<tr><td>wtdbg2</td><td>Nanopore</td><td>wtdbg2 -x ont -g 100m -i ont_data -fo out
wtpoa-cns -i out.ctg.lay.gz -o out.ctg.fa
minimap2 -x map-ont -a out.ctg.fa ont_data | samtools sort > out.ctg.bam
samtools view out.ctg.bam | wtpoa-cns -d out.ctg.fa -i - -fo assembly.fasta</td></tr>
<tr><td>HaploMerger2</td><td>-</td><td>samtools faidx assembly.fasta
BuildDatabase -name asm.db -engine ncbi assembly.fasta
RepeatModeler -engine ncbi -database asm.db
RepeatMasker -e ncbi -lib consensi.fa -xsmall assembly.fasta
run_all.batch</td></tr>
<tr><td>purge_dups</td><td>PacBio</td><td>echo pb_data > input.fofn
pd_config.py assembly.fasta input.fofn
run_purge_dups.py config.json purge_dups_bin species_id</td></tr>
<tr><td>purge_dups</td><td>Nanopore</td><td>echo ont_data > input.fofn
pd_config.py assembly.fasta input.fofn
run_purge_dups.py config.json purge_dups_bin species_id</td></tr>
<tr><td>purge_haplotigs</td><td>PacBio</td><td>minimap2 -ax map-pb assembly.fasta ont_data --secondary=no > aligned.bam
samtools sort -o ali.sorted.bam -T tmp.ali aligned.bam
samtools index ali.sorted.bam
samtools faidx assembly.fasta
purge_haplotigs hist -b ali.sorted.bam -g assembly.fasta
purge_haplotigs cov -i ali.sorted.bam -l L -m M -h H -o cov_stats.csv
purge_haplotigs purge -g assembly.fasta -c cov_stats.csv -o assembly.purged.fasta</td></tr>
<tr><td>purge_haplotigs</td><td>Nanopore</td><td>minimap2 -ax map-ont assembly.fasta ont_data --secondary=no > aligned.bam
samtools sort -o ali.sorted.bam -T tmp.ali aligned.bam
samtools index ali.sorted.bam
samtools faidx assembly.fasta
purge_haplotigs hist -b ali.sorted.bam -g assembly.fasta
purge_haplotigs cov -i ali.sorted.bam -l L -m M -h H -o cov_stats.csv
purge_haplotigs purge -g assembly.fasta -c cov_stats.csv -o assembly.purged.fasta</td></tr>
<tr><td>BBtools</td><td>-</td><td>reformat.sh in=long_reads_data out=subset_data samplebasestarget=number_of_bases</td></tr>
<tr><td>BUSCO</td><td>-</td><td>busco -i assembly.fasta -o busco_output -l metazoa_odb10 -m genome</td></tr>
<tr><td>KAT</td><td>Illumina</td><td>kat comp -o kat_output 'end1.fastq end2.fastq' assembly.fasta</td></tr>
<tr><td>tinycov</td><td>Nanopore</td><td>minimap2 -x map-ont -a assembly.fasta ont_data | samtools sort > aligned.bam
tinycov covplot -r 20000 -t cov.txt aligned.bam</td></tr>
<tr><td>tinycov</td><td>PacBio</td><td>minimap2 -x map-pb -a assembly.fasta pb_data | samtools sort > aligned.bam
tinycov covplot -r 20000 -t cov.txt aligned.bam</td></tr>
<tr><td>HapPy</td><td>Nanopore</td><td>minimap2 -x map-ont -a assembly.fasta ont_data | samtools sort > aligned.bam
HapPy.py depth aligned.bam out_dir
HapPy.py estimate out_dir/aligned.bam.hist</td></tr>
<tr><td>HapPy</td><td>PacBio</td><td>minimap2 -x map-pb -a assembly.fasta pb_data | samtools sort > aligned.bam
HapPy.py depth aligned.bam out_dir
HapPy.py estimate out_dir/aligned.bam.hist</td></tr>
<tr><td>time</td><td>-</td><td>/usr/bin/time -v -o time_output.txt</td></tr>
</table>

Table S8. Long-read and short-read datasets used in the study.

Data type	Minimum length	Total data	N50
PacBio	-	23.5 Gb	11.6 kb
	15 kb	4.7 Gb	17.6 kb
Nanopore	-	17.5 Gb	18.8 kb
	30 kb	5.7 Gb	51.8 kb
Illumina 2*250 bp	30 bp	11.4 Gb	250 bp
